# Programmable artificial RNA condensates in mammalian cells

**DOI:** 10.1038/s41565-026-02164-7

**Published:** 2026-04-29

**Authors:** Shiyi Li, Yuna Kim, Kevin Wang, Eric John Payson, Anli A. Tang, Maria Villalba Nieto, Dino Osmanovic, Madison Yang, Diego Dilao, Alexandra Bermudez, Wen Xiao, Melody M. H. Li, Neil Y. C. Lin, Kathrin Plath, Douglas L. Black, Elisa Franco

**Affiliations:** 1 Department of Bioengineering, University of California, Los Angeles, Los Angeles, CA USA; 2 Department of Mechanical and Aerospace Engineering, University of California, Los Angeles, Los Angeles, CA USA; 3 Department of Microbiology, Immunology, and Molecular Genetics, University of California, Los Angeles, Los Angeles, CA USA; 4 Department of Chemistry and Biochemistry, University of California, Los Angeles, Los Angeles, CA USA; 5 Department of Molecular, Cell and Developmental Biology, University of California, Los Angeles, Los Angeles, CA USA; 6 Department of Biological Chemistry, David Geffen School of Medicine, University of California, Los Angeles, Los Angeles, CA USA

**Keywords:** Nanostructures, RNA nanotechnology

## Abstract

Artificial biomolecular condensates have emerged as powerful tools for controlling cellular behaviour. Here we introduce a method to build artificial condensates within living mammalian cells by designing modular RNA motifs composed of a single short RNA strand. These condensates emerge spontaneously, creating RNA-rich compartments that remain separated from their surrounding environment. The RNA sequences include stem–loop domains that fold as the RNA is transcribed, and then condense in the nucleus and cytoplasm through loop–loop interactions. These sequences can be optimized and diversified, enabling the generation of distinct, non-mixing condensate populations and the programmable control of their subcellular localization. The RNA motifs can also be modified to recruit small molecules, proteins and RNA molecules in a sequence-specific manner to the RNA-rich phase. By introducing RNA linkers, we can build condensates with multiple subcompartments, whose organization can be controlled by tuning the linker stoichiometry. These artificial condensates provide a versatile platform for studying and manipulating molecular functions inside living cells.

## Abstract (web summary)

Modular RNA nanostar motifs spontaneously assemble into programmable synthetic condensates in the nucleus and cytoplasm, with RNA design controlling localization, sequence-specific target recruitment and orthogonal assembly.

## Main

Biomolecular condensates are associated with a multitude of processes in living cells, including gene expression, metabolism and diseases^1^. For this reason, there is growing interest in methods for producing artificial condensates with controllable composition, viscoelastic properties and subcellular location, as a means for manipulating cellular functions^2^. One approach to building artificial condensates is to establish weak interactions among engineered proteins that carry intrinsically disordered domains^3,4^. Similarly, RNA molecules designed to include multiple, short sequence repeats yield artificial RNA condensates^5–8^. As weak promiscuous bonds make it difficult to build condensates with a compositional identity and exclusive functions, a promising alternative approach is to take advantage of multivalent molecules with site-specific interactions^9,10^.

**Fig. 1:**
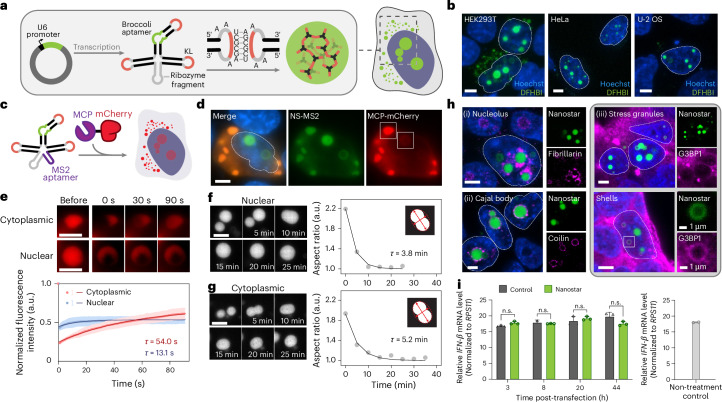
Circularized RNA nanostars generate condensates in multiple mammalian cell lines. **a**, RNA nanostars were expressed in mammalian cells from transfected Tornado plasmids, yielding circularized RNA motifs that form condensates through KL interactions. **b**, Representative confocal microscopy images showing RNA condensate formation in HEK293T, HeLa and U-2 OS cells. The nuclei of condensate-expressing cells are outlined. **c**, A schematic illustrating the recruitment of MCP-mCherry to RNA nanostars through the incorporation of an MS2 aptamer as an additional arm. **d**, Split channel fluorescent images of a HEK293T cell cotransfected with MCP-mCherry and MS2-nanostar. Two representative condensates analysed by FRAP in **e** are highlighted. **e**, Representative fluorescence images (top) and pixel intensity profile (bottom) at different time points after photobleaching. Blue and orange dots represent the mean pixel intensity within the bleached region at each time point. Error bars indicate standard deviation. Dark blue and red lines show exponential fits with time constant *τ*. Means and standard deviations were calculated from three fields of view, each from an independent replicate. **f**,**g**, Fluorescence images (left) and time-dependent changes in aspect ratio (right) demonstrating the coalescence of nuclear (**f**) and cytoplasmic (**g**) condensates in HEK293T cells expressing Broccoli-labelled nanostars. Aspect ratios (grey dots) were calculated as the ratio of major to minor axes from best-fit ellipses. Dashed lines indicate exponential fits with decay constant *τ*. **h**, Colocalization of RNA condensates with endogenous membraneless organelles in HEK293T cells. Cell nuclei (blue), RNA condensates (green) and canonical marker proteins for each indicated organelle labelled by immunostaining (magenta) are shown. **i**, Quantitative PCR analysis of *IFN-beta* mRNA expression in nanostar-expressing cells (green), Lipofectamine-treated cells (dark grey) and untreated cells (light grey) at the indicated time points post-transfection. Statistical analysis using two-way ANOVA followed by multiple comparison tests showed no significant difference. All cellular expression data were collected 48 h post-transfection, except for the time-lapse experiments. Images in **b** are *z*-projections from confocal stacks. All experiments were performed in three independent replicates, and representative images are shown. Scale bars, 5 μm. n.s., not significant. Source data

We and others recently described in vitro assembly of RNA condensates from short single strands of RNA (100–200 nucleotides long) that operate as multivalent particles^11,12^. These structural motifs, termed single-stranded RNA (ssRNA) nanostars, consist of at least three stem–loops that fold during transcription, functioning as ‘arms’ (Fig. 1a). The arms interact through sequence-specific binding of their loop domains, known as kissing loops (KLs), which are typically palindromic and identical on each arm. KLs can be designed to be non-interacting, so distinct nanostars can produce condensates that do not mix^11,12^. The modular design of nanostars allows for the addition of domains for the recruitment of small molecules and proteins^11,12^. Here, we demonstrate that ssRNA nanostars can generate condensates within living mammalian cells with controlled mixing patterns, and that the interactions and localization of these condensates can be tuned by modifying the nanostar design.

## RNA nanostars produce condensates that recruit target molecules

We produced ssRNA nanostars in living cells using the Tornado expression system^13^ for spontaneous RNA circularization that extends the RNA’s half-life (Supplementary Figs. 1 and 2 and Supplementary Table 1). We started with a nanostar design that carries three 15-nucleotide (nt) arms, each with a 6-nt KL UCGCGA (variant A), as shown in Fig. 1a (design 15nt-3A-Br). We included the Broccoli aptamer in one of the arms, to visualize the expressed RNA in cells by adding its fluorogenic ligand 3,5-difluoro-4-hydroxybenzylidene imidazolinone (DFHBI) to the media^14^. This nanostar design generates condensates during in vitro transcription at a constant temperature (Supplementary Fig. 3), and we observed comparable structures forming in transfected HEK293T, HeLa and U-2 OS cells (Fig. 1b and Supplementary Figs. 4 and 5). Further experiments were done in HEK293T cells, which show higher expression levels owing to the presence of the SV40 large T antigen^15,16^. Flow cytometry experiments confirmed that expression of circularized Broccoli alone results in lower granularity when compared with nanostar-expressing cells (Supplementary Fig. 6). Microscopy further confirmed diffuse fluorescence in the cytoplasm, with few puncta in the nucleus, and shells that may correspond to circularized RNA colocalized with other cellular components^13,17^ (Supplementary Fig. 7a). Replacement of at least one KL with a polyA sequence disrupts the formation of full droplets, although shells persist, confirming that condensation is driven by loop–loop interactions (Supplementary Fig. 7b).

RNA nanostars can recruit ‘guest’ molecules through embedded aptamers. We demonstrate this using the MS2 aptamer to recruit an MCP-tagged reporter^18^. We cotransfected two plasmids into cells, one encoding the modified MS2-RNA nanostar and the other encoding the MCP-mCherry guest (Fig. 1c and Supplementary Figs. 8 and 9), and measured the colocalization of fluorescent signals. Live-cell imaging confirmed successful mCherry recruitment into RNA condensates (Fig. 1d and Supplementary Fig. 10). By contrast, nanostars lacking the MS2 domain did not affect the spatial localization of mCherry (Supplementary Fig. 9).

## Diffusivity and viscosity of RNA condensates

Using fluorescence recovery after photobleaching (FRAP), we estimated the diffusion timescale for the mCherry guest protein. Cytoplasmic condensates exhibit a recovery time constant of 54 s, while nuclear condensates show a faster but minimal recovery (Fig. 1e and Supplementary Fig. 11), probably because of the lower level of mCherry, which lacks a nuclear localization signal. The abundance of mCherry correlated with higher percentage of recovery, presumably because guest-molecule recruitment hinders nanostar interactions and increases guest diffusivity (Supplementary Fig. 12). FRAP analysis of nuclear and cytoplasmic condensates using Broccoli reveals faster recovery, *τ* = 7.8 s and *τ* = 10.2 s (Supplementary Fig. 13a–d), respectively, and *τ* = 18.2 s in vitro (Supplementary Fig. 14a). Because small-molecule ligands bind their RNA aptamer non-covalently (Broccoli *K*_d_ ≈ 300 nM), the observed recovery is probably dominated by dynamic ligand exchange rather than RNA diffusion. Nuclear condensates labelled with Pepper^17,19^, another fluorogenic aptamer that produces red fluorescence upon the addition of (4-((2-hydroxyethyl)(methyl)amino)-benzylidene)-cyanophenylacetonitrile 620 (HBC620), showed distinct recovery with a larger fitted time constant of 79.5 s, consistent with a lower *K*_d_ ≈ 3.5 nM (ref. ^20^) when compared with Broccoli (Supplementary Fig. 13e,f).

Fusion of RNA condensates in living cells proceeded with a time constant of *τ* = 3.8 min for a nuclear event, and *τ* = 5.2 min for a cytoplasmic event (Fig. 1f,g and Supplementary Figs. 16–19). Nuclear events appeared faster than cytoplasmic events, potentially reflecting differences in environment. Although faster in cells than in vitro (*τ* ≈ 16 h) (Supplementary Fig. 15), the fusion of RNA condensates remains slower than that of P granules and stress granules, which relax within seconds^21,22^, and is comparable to nucleolar dynamics^23^. Together, FRAP and fusion results indicate that RNA condensates are relatively viscous. We observed a nuclear ‘splitting’ event (Supplementary Fig. 19), suggesting influence from other cellular components.

## RNA condensates interact with nuclear organelles but do not elicit an immune response

Immunostaining for fibrillarin indicates an accumulation of RNA nanostars in the nucleolar region (Fig. 1h(i) and Supplementary Fig. 20a). In these cells, fibrillarin localized at the condensate surface but remained excluded from the condensates. Some RNA nanostar variants might be favoured for nucleolar localization (Supplementary Fig. 20b). Similarly, coilin aggregates on the condensates surface, suggesting the recruitment of Cajal bodies (Fig. 1h(ii) and Supplementary Fig. 21). Cells producing high levels of RNA condensates showed increased stress granule formation, as indicated by G3BP1 staining (Fig. 1h(iii), top, and Supplementary Fig. 22). Colocalization of G3BP1 and nanostars was only observed in a few cells with high cytoplasmic RNA condensate formation^24,25^ (Supplementary Fig. 22e). In some nuclei, G3BP1 colocalized on the outside of shells, but not with condensates (Fig. 1h(iii), bottom, and Supplementary Fig. 23). Shells were observed in other systems producing circularized RNA, indicating that they are not specific to nanostar condensates^17^ (Supplementary Figs. 7 and 23). More work is needed to determine whether stress granules in nanostar-expressing cells arise owing to specific RNA–protein interactions, or because of metabolic stress caused by elevated cytoplasmic RNA levels^24–27^. We found no colocalization with P-bodies and nuclear speckles (Supplementary Fig. 24). No immunogenic response was observed in transfected cells, as indicated by comparable mRNA expression levels of interferon beta 1 (*IFNB1*) and the IFN-stimulated genes *ISG15* and *IFIT1* in cells with and without condensates (Fig. 1i, Supplementary Fig. 25 and Supplementary Table 4).

## RNA nanostar design determines the subcellular localization of condensates

**Fig. 2:**
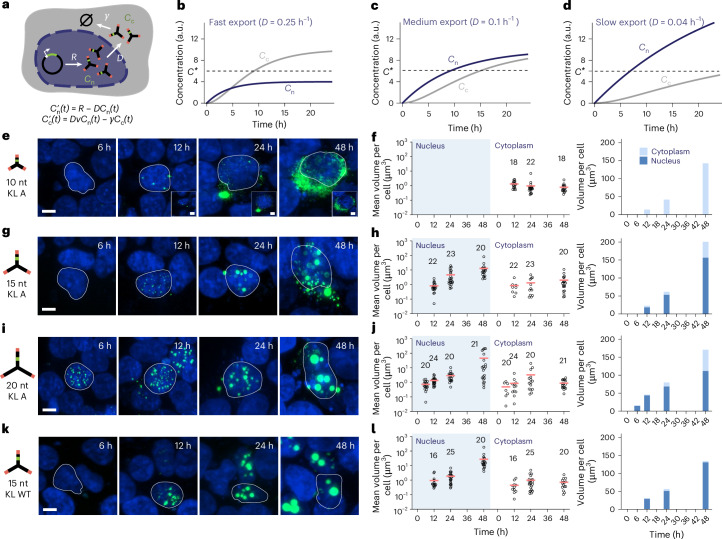
Nuclear and cytoplasmic condensate formation depends on nanostar design. **a**, A model schematic depicting parameters determining nanostar concentration in the nucleus (*C*_n_) and cytoplasm (*C*_c_). *R* denotes the transcription rate, *D* the nuclear export rate and *γ* the degradation rate. *ν* represents the nuclear-to-cytoplasm volume ratio (*V*_n_:*V*_c_). ∅ indicates RNA degradation products. Phase separation occurs when concentration exceeds the critical concentration (C*). **b**–**d**, Simulated concentration profiles for nuclear (*C*_n_) and cytoplasmic (*C*_c_) compartments at fast (**b**), medium (**c**) or slow (**d**) export rates (*D*), with other parameters held constant (*R* = 1, *γ* = 0.2 and *ν* = 2). Faster export favours cytoplasmic accumulation, whereas slower export promotes nuclear enrichment. **e**,**g**,**i**,**k**, Representative confocal *z*-projections showing condensate growth over 48 h for nanostars with KL A and 10-nt (**e**), 15-nt (**g**) and 20-nt (**i**) arms, and for nanostars with KL WT and 15-nt arms (**k**). Nuclei are stained with Hoechst (blue) and nanostars with DFHBI (green). The same cell is outlined across time points for each design. Insets in **e** show single midplane sections confirming cytoplasmic localization. Additional midplane images are provided in Supplementary Fig. 29. **f**,**h**,**j**,**l**, Quantification of condensate formation from nanostars with KL A and 10-nt (**f**), 15-nt (**h**) and 20-nt (**j**) arms, and for nanostars with KL WT and 15-nt arms (**l**). Dot plots (left) show the temporal evolution of mean condensate volume per cell in the nucleus and cytoplasm; numbers above indicate the number of analysed cells at each time point across three independent replicates. Bar plots (right) show total condensate volume per cell averaged across analysed cells. Scale bars, 5 μm. Source data

Local concentration of nanostars is determined by the relative speed of transcription in the nucleus, export from the nucleus and degradation. These processes crucially affect condensate formation, which occurs only if the nanostar concentration exceeds a critical value C* (ref. ^28^). We illustrate this through a simple compartment model (Fig. 2a), in which nanostars are produced at a rate of *R* inside the nucleus, exported to the cytoplasm at a constant rate *D* and degraded in the cytoplasm at rate *γ*

1 $${C}_{{\rm{n}}}^{{\prime} }(t)=R-{{DC}}_{{\rm{n}}}(t).$$

2 $${C}_{{\rm{c}}}^{{\prime} }(t)=D\,\nu {C}_{{\rm{n}}}(t)-{\rm{\gamma }}{C}_{{\rm{c}}}(t).$$

Here, *C*’_n_(*t*) and *C*’_c_(*t*) denote the time derivatives of the nanostar concentrations in the nucleus (*C*_n_) and cytoplasm (*C*_c_), *ν* is a correction term accounting for the change in volume ratio *V*_n_:*V*_c_. The production rate *R* depends primarily on the amount of plasmids delivered into each cell, and the degradation rate *γ* should only depend on the nanostar concentration. By contrast, the export constant *D*, governed by the size-sensitive permeability of the nuclear pore, can be dramatically affected by structural design features of nanostars. Depending on the value of *D*, the critical concentration may never be reached, or can be reached at different times in the nucleus and in the cytoplasm (Fig. 2b–d). By systematically changing the nanostar size and intermolecular affinity, one could modulate where and when condensates form^29^.

To test this model, we first modified the nanostar arm length (10, 15 and 20 nt)^11^, and measured the nuclear and cytoplasmic volume of RNA condensates at 6, 12, 24 and 48 h post-transfection^30^ (Fig. 2e–j and Supplementary Figs. 26–28). We found that 10-nt arm nanostars (arm length 3.4 nm, molecular weight (MW) 56.8 kDa) form condensates exclusively in the cytoplasm as they probably diffuse quickly through the nuclear pore complex (Fig. 2e,f), demonstrating a similar behaviour as simulated in Fig. 2b. These nanostars do not colocalize with nucleoli nor with Cajal bodies (Supplementary Figs. 20b and 21b). Nanostars with a 15-nt arm (arm length 5.1 nm, MW 66.3 kDa) and a 20-nt arm (arm length 6.8 nm, MW 75.8 kDa), having sizes close to the nucleus’ permeability barrier, form condensates inside the nucleus first, and the cytoplasmic fraction increases over time (Fig. 2g–j). Nanostars with 20-nt arms condensed at an earlier time point, reflecting a dependence of growth rate on nanostar size^31^. In addition to depending on arm length, the nanostar export rate could also be impacted by the rate of assembly. To test this we changed the 15-nt arm nanostar from KL A (UCGCGA) to the strongest possible variant, KL WT (GCGCGC)^32^ and observed an increase in the nuclear condensate fraction (Fig. 2k,l).

**Fig. 3:**
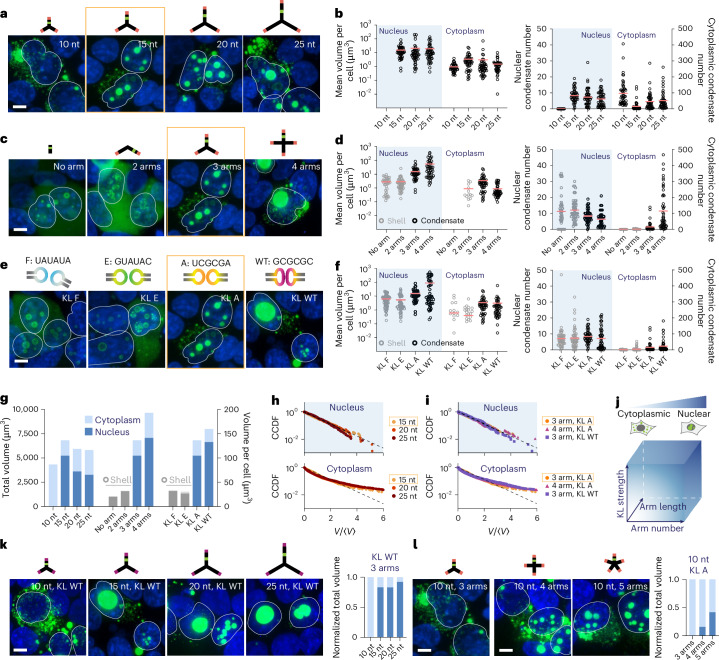
Engineering condensate volume distribution and localization through nanostar design. **a**,**c**,**e**, Representative confocal *z*-projections showing condensates formed by RNA nanostar variants with different arm lengths (**a**), arm numbers (**c**) or KL sequences (**e**). Nuclei are stained with Hoechst (blue) and nanostars with DFHBI (green). The nuclei of condensate-expressing cells are outlined. Yellow boxes highlight the same variant across panels. **b**,**d**,**f**, Quantification of condensate formation from nanostars with different arm lengths (**b**), arm numbers (**d**) or KL sequences (**f**). Mean condensate volume per cell in the nucleus and cytoplasm (log scale) (left). Number of condensates per cell in the nucleus and cytoplasm (right). Each dot represents one cell; red lines indicate the mean. **g**, Total condensate volume across 50 analysed cells (left axis) and mean volume per cell (right axis) for the indicated designs. **h**,**i**, CCDFs of condensate volumes. Nuclear condensates are well described by an exponential distribution (dashed line), whereas cytoplasmic condensates exhibit sub-exponential distribution across variants differing in arm length (**h**), arm number or KL sequence (**i**). Condensates were segmented in three dimensions, normalized to the mean condensate volume of each cell, pooled across cells and plotted collectively. Each dot represents a single condensate. **j**, A schematic summarizing the relationship between nanostar design parameters and condensate localization. Increased KL strength, arm length or arm number promotes nuclear enrichment. **k**,**l**, Representative confocal images (left) showing enhanced nuclear localization with increased arm length (**k**) or arm number (**l**). The quantification of normalized total condensate volume from 50 cells (right) shows an increased nuclear fraction (dark blue) relative to cytoplasm (light blue). All experiments were performed in three independent replicates. Scale bars, 5 μm. Source data

We then elucidated how design parameters influence condensate localization, abundance and morphology, by systematically varying size (arm length), valency (number of arms) and avidity (KL strength) of the nanostars^31^ (Fig. 3). For each variant, we measured corresponding condensate volume, number and cellular localization 48 h after transfection (Supplementary Figs. 29–33). Consistent with Fig. 2, nanostars with 10-nt arms formed condensates only in the cytoplasm, while nanostars with 15–25-nt long arms also generated fewer and larger condensates in the nucleus (Fig. 3a,b). Next, we varied the nanostar arm number between two and four (Fig. 3c,d). Nanostars with valency less than three only formed shells. Replacing KLs with polyA sequences yielded similar effects as deleting arms (Supplementary Fig. 7). Increasing arm number yielded fewer but larger condensates in the nucleus, probably due to accelerated nuclear aggregation, and correspondingly, more small puncta in the cytoplasm, probably due to enhanced nucleation. Finally, we investigated the impact of KL strength on condensate formation across four variants, with GC content spanning from 0% to 100% (Fig. 3e,f and Supplementary Table 2). The strongest variant (WT, GCGCGC) yielded large condensates in the nucleus and a higher number of smaller puncta in the cytoplasm, resembling the behaviour of the four-arm nanostar. By contrast, weak KL variants E (GUAUAC) and F (UAUAUA) primarily formed nuclear shells. Overall, larger and fewer condensates accumulated in the nucleus as we increased arm number and strength of the KLs (Fig. 3g, Supplementary Fig. 30 and Supplementary Table 3).

Across designs, normalized volume complementary cumulative distribution functions (CCDFs)^33^ show that nuclear condensates follow an exponential distribution, while cytoplasmic condensates are sub-exponential (Fig. 3h,i). This difference may be because of faster condensate birth inside the nucleus when compared with the cytoplasm^33^. No apparent deviation in the normalized CCDF was observed across designs. Similar trends are observed in the CCDF of condensate volume measured at different time points, from the experiments reported in Fig. 2 (Supplementary Fig. 31).

Overall, these results show that RNA nanostar design provides means to tune the nuclear-to-cytoplasmic distribution of condensates. The proportion of nuclear condensates increases with stronger KLs, more arms and longer arms (Fig. 3j and Supplementary Fig. 32). We further validated this programmable control by (1) maximizing nuclear localization and (2) relocating cytoplasmic-only condensates into the nucleus through sequence modifications (Fig. 3k,l and Supplementary Fig. 33). First, we introduced the KL WT to enhance interaction strength and observed almost exclusively nuclear condensates for the 25-nt nanostar (Fig. 3k). Next, we increased the arm number from three to five, reasoning that higher valency and molecular size might synergistically promote nuclear localization. As expected, 10-nt nanostars with more arms formed nuclear condensates whose size and number scaled with arm number (Fig. 3l).

Collectively, these experiments demonstrate that RNA nanostars are a robust motif for building cellular condensates, and that structural and sequence variations enable control over their localization and size. Partition coefficient analysis across all designs confirmed higher nuclear enrichment when compared with cytoplasmic condensates, while shell-forming constructs consistently displayed low partition coefficients (Supplementary Fig. 34). We verified that variations to the nanostar design do not compromise their ability to recruit target proteins (Supplementary Fig. 35a), but the diffusivity of guest molecules might vary following the change in the interaction of nanostars (Supplementary Fig. 35b). Finally, we observed that cells expressing RNA nanostars exhibit an enlarged nucleus, probably as a consequence of increased local osmotic pressure (Supplementary Figs. 36 and 37).

## Sequence design produces diverse condensates with tunable interactions and cellular localization

**Fig. 4:**
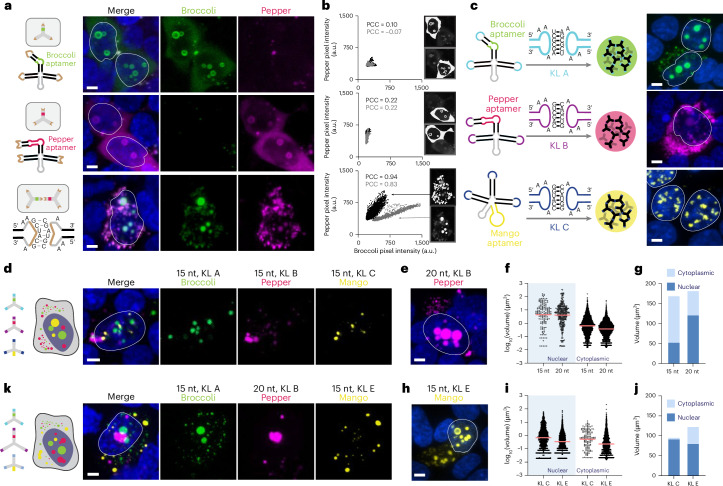
Nanostar KL sequences determine condensate miscibility. **a**, A schematic of nanostars labelled with Broccoli or Pepper that interact through complementary, non-palindromic KLs. Fluorescence micrographs show condensate formation upon coexpression of both nanostars. **b**, Scatter plots of Broccoli and Pepper pixel intensities within the indicated regions of interest, with corresponding PCCs. The lower panel compares nuclear and cytoplasmic condensates, which exhibit distinct correlation patterns. **c**, A schematic of nanostars bearing KLs A, B or C, labelled with Broccoli, Pepper or Mango aptamers, and representative fluorescence images of the resulting condensates. **d**, Cotransfection of 15-nt arm nanostars carrying distinct KLs (A, B and C) produces spatially segregated condensates that do not mix. **e**,**h**, Confocal images of cells expressing modified nanostars with altered arm length (**e**, Pepper tagged) or KL strength (**h**, Mango tagged) to modulate subcellular localization. **f**,**i**, Dot plots showing the distribution of nuclear and cytoplasmic condensate volumes (log scale) forming from Pepper-tagged (**f**) or Mango-tagged (**i**) nanostars. Each dot represents one condensate; red lines indicate the mean. **g**,**j**, Bar charts comparing the average condensate volume in the nucleus and cytoplasm forming from Pepper-tagged (**g**) or Mango-tagged (**j**) nanostars. **k**, Reprogrammed subcellular localization of distinct condensates using modified Pepper- and Mango-tagged nanostars while maintaining the Broccoli design. Cells were stained with Hoechst (nuclei), DFHBI (Broccoli), HBC620 (Pepper), and TO1-B (Mango). Nuclei are outlined. All experiments were performed in three independent replicates. Images are confocal *z*-projections. Scale bars, 5 μm. Source data

RNA nanostars can be created to have diverse KL domains (Fig. 4 and Supplementary Fig. 38). First, we tested a two-nanostar system with non-palindromic, complementary KLs^11^ (Fig. 4a), each labelled with distinct fluorogenic aptamers, Broccoli and Pepper. No condensation was observed when only one of these two nanostars was expressed (Fig. 4b, top and middle). When both nanostars are coexpressed, condensates show colocalized Broccoli and Pepper fluorescence with a high Pearson correlation coefficient (PCC) (Fig. 4b, bottom). Distinct slopes in the fluorescence scatter plot suggest different Broccoli-to-Pepper ratios in the cytoplasm and nucleus, probably due to the influence of aptamers on nanostar folding and nuclear export speed.

Leveraging the sequence-specificity of loop–loop interactions, RNA nanostars can be optimized to generate distinct, non-interacting condensates. To illustrate this we designed three nanostars^12^, each carrying distinct palindromic KLs (A, B and C) and different fluorogenic aptamers, Broccoli, Pepper and Mango (produces yellow fluorescence upon the addition of TO1-Biotin (TO1-B))^34^. Each nanostar generated condensates, with differences in their cellular localization depending on the aptamer reporter (Fig. 4c and Supplementary Fig. 39). While the distribution of Broccoli- and Pepper-carrying nanostars remained consistent with variants described earlier, Mango-carrying nanostars with KL C primarily yielded nuclear condensates, as the Mango aptamer loop might enhance nanostar interactions and promote nuclear aggregation. Simultaneous transfection of plasmids, each carrying one of the nanostars, resulted in distinct, non-mixing condensates (Fig. 4d and Supplementary Fig. 40), which maintained the subcellular localization observed when expressed individually.

Taking advantage of the design guidelines identified in Figs. 2 and 3, we then sought to shift the cellular localization of non-mixing condensates. To shift Pepper-tagged condensates to the nucleus, we extended the nanostar arm length from 15 nt to 20 nt. With this change, we observed a higher nuclear-to-cytoplasmic volume ratio (Fig. 4e–g). Enhancing KL strength was a less effective strategy (Supplementary Fig. 42a–c). To localize Mango-tagged nanostars more to the cytoplasm, we adopted weaker KLs, switching from design C (GGUACC) to design E (GUAUAC). This change successfully increased the cytoplasmic-to-nuclear condensate volume (Fig. 4h–j). Adopting KL F (AUAUAU) yielded only nuclear shells (Supplementary Fig 42d). Cotransfection of the new plasmid triplet resulted in distinct condensates with the expected changes in subcellular distribution (Fig. 4k). Taken together, these results indicate that the principles governing the interaction specificity, morphology and cellular localization of condensates remain valid across variants; however, the efficiency in manipulating condensate properties is affected by details of nanostar and aptamer sequence.

**Fig. 5:**
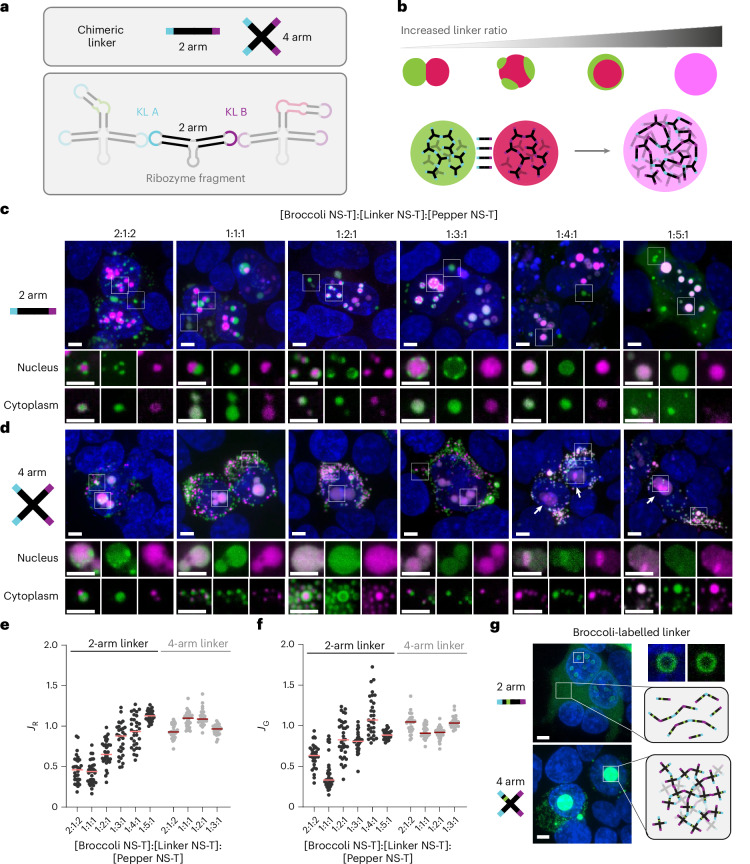
Chimeric linker nanostars at different ratios yield condensates with programmable sub-compartmentalization. **a**, A schematic of two-arm and four-arm chimeric linker nanostars. **b**, Increasing the ratio of chimeric linker nanostars relative to distinct nanostars enhances condensate mixing. **c**,**d**, Confocal micrographs of cells expressing nanostars at varying plasmid ratios ([Broccoli nanostar plasmid (NS-T)]:[Linker NS-T]:[Pepper NS-T]) using two-arm (**c**) or four-arm (**d**) linkers. Top panels show larger fields of view (*z*-projections) and bottom panels show zoomed-in sections. White arrows indicate non-homogeneous mixing, probably owing to clustering of the four-arm linkers. **e**,**f**, Quantification of mixing for two-arm and four-arm linkers. For two-arm linkers, mixing indices *J*_R_ (**e**) and *J*_G_ (**f**) increase with linker ratio. Four-arm linkers exhibit higher mixing indices consistent with increased valency. **g**, Broccoli-labelled two-arm linkers do not form condensates on their own, whereas four-arm linkers self-assemble into condensates. Cells were stained with Hoechst (nuclei), DFHBI (Broccoli) and HBC620 (Pepper). Images were acquired 48 h after transfection. All experiments were performed in three independent replicates. Scale bars, 5 μm. Source data

We then demonstrated how to systematically colocalize non-mixing condensates (20 nt, KLs A and B, labelled with Broccoli and Pepper, respectively) through the production of RNA linkers carrying both KLs^12,35^ (Fig. 5 and Supplementary Figs. 43 and 44). We tested two- and four-arm linkers (Fig. 5a). The colocalization level of distinct condensates depends on the ratio of the linker plasmid concentration relative to the nanostar plasmid concentration (Fig. 5b). At levels of nanostar:two-arm-linker:nanostar-plasmid ratios up to 1:3:1, nuclear Broccoli- and Pepper-carrying condensates interacted while remaining distinct, producing Janus-like morphologies (Fig. 5c, top row). At higher ratios, they completely mixed. We observed similar results for the four-arm linker (Fig. 5d, top row); however, a 1:2:1 ratio of nanostar:four-arm-linker:nanostar was sufficient to produce mixed condensates in the nucleus. Because each linker now carried twice as many arms, this result was consistent with the two-arm linker, which yielded mixed condensates at a 1:4:1 ratio. In the cytoplasm, however, two-arm linkers produced mixed condensates at lower plasmid ratios when compared with four-arm linkers, presumably because the smaller two-arm motifs are exported faster from the nucleus and their concentrations increase more rapidly in the cytoplasm (Fig. 5c,d, bottom row).

The degree of mixing between the two phases of nuclear condensates was quantified through mixing indices *J*_R_ and *J*_G_ that measured the normalized fraction of red in green, and green in red condensates^12^ (Fig. 5e,f). Compared with *J*_R_, index *J*_G_ showed more fluctuation owing to a lower signal-to-noise ratio of Broccoli compared with Pepper (Supplementary Fig. 45). To our surprise, Broccoli-labelled four-arm linkers formed condensates when expressed individually (Fig. 5g), suggesting that the non-homogeneous mixing observed in Fig. 5d (white arrows) probably resulted from the local clustering of non-fluorescently labelled linkers.

## Recruitment of target RNAs in *trans*

**Fig. 6:**
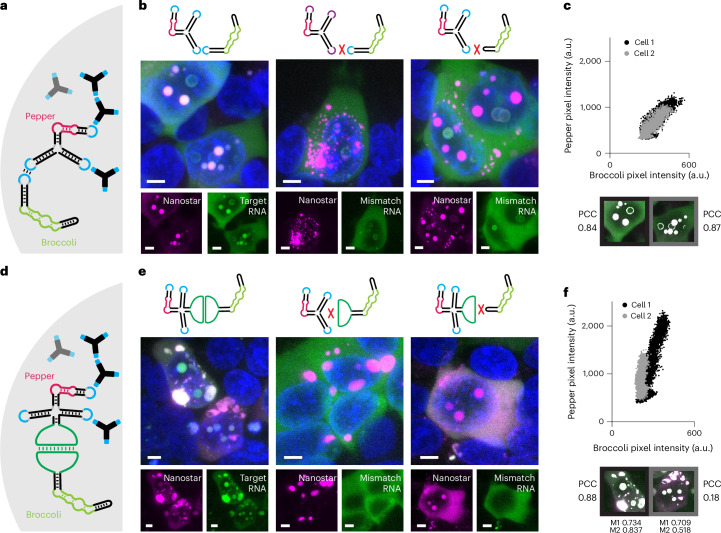
RNA nanostar condensates recruit cellular RNA molecules through sequence-specific interactions. **a**, A schematic illustrating the recruitment of Broccoli-labelled RNA molecules to condensates formed by Pepper-labelled nanostars through shared KL sequences. **b**, Confocal micrographs showing colocalization only when both the nanostar and target RNA contain matching KL sequences. **c**, Scatter plots of Broccoli and Pepper pixel intensities within the indicated regions of interest, with corresponding PCCs. **d**, A schematic illustrating the recruitment of Broccoli-labelled target RNAs to condensates formed by Pepper-labelled nanostars engineered with a complementary recruitment domain. **e**, Confocal micrographs showing colocalization only when both the nanostar and target RNA contain matching recruitment domains. **f**, Scatter plots of Broccoli and Pepper pixel intensities within the indicated regions of interest, with corresponding PCC values. In cell 2, low target RNA expression reduces signal-to-noise and lowers the PCC despite visible overlap. Manders’ overlap coefficients are provided to quantify the fraction of overlapping signal. Cells were stained with Hoechst (nuclei), DFHBI (Broccoli) and HBC620 (Pepper). Control PCC analyses are shown in Supplementary Fig. 46. All experiments were performed in three independent replicates. Images are confocal *z*-projections. Scale bars, 5 μm. Source data

One unique advantage of RNA nanostars is their ability to recruit specific target RNA through base pairing. To demonstrate this, we first built a model RNA target that included a single stem–loop domain with a KL complementary to the nanostar KL. This target was successfully colocalized with the condensates (Fig. 6a), as indicated by the PCC and the partition coefficient (Fig. 6c and Supplementary Fig. 46i). By contrast, we found no colocalization when the correct KL sequence was missing on either the nanostar or the target RNA (Fig. 6b and Supplementary Fig. 46d,e). While successful, this approach requires modification of the target RNA, which may be undesirable. To bypass this constraint, we modified nanostars to carry a dedicated hybridization domain complementary to the target RNA (Fig. 6d). This is a sequence-specific recruitment strategy applicable in principle to any RNA transcript, provided that the intermolecular hybridization outcompetes local secondary structures. As a proof of concept, we used an arbitrary 21-nt sequence as the recruitment domain. Colocalization was observed only when this domain matched the target RNA sequence (Fig. 6e,f and Supplementary Fig. 46f–h,j). To quantify colocalization, we provided Manders’ overlap coefficients, which assess the fraction of overlapping independent of signal intensities, as well as PCC values, whose low values are because of the low expression levels of target RNA molecules. Although the long hybridization domain slightly reduced condensate formation, probably owing to steric hindrance, recruitment was unaffected, and condensation could be improved by increasing the nanostar-to-target RNA ratio (Supplementary Fig. 46c).

## Conclusion

We have demonstrated a simple yet highly programmable strategy to build RNA condensates in living mammalian cells from short ssRNAs that fold into star-shaped motifs. Leveraging advances in DNA and RNA nanotechnology^35–40^, our work establishes that sequence-specific RNA interactions can act as a structural and functional driver of condensate formation, enabling precise control of condensate material properties and cellular localization through rational sequence design^2,41–46^.

A major contribution of this study is the demonstration that synthetic RNA condensates can spatially localize small molecules, proteins and other RNAs through sequence design independently of any cellular localization signals and allows tuning of the nuclear-to-cytoplasmic ratio. Because most cellular reactions occur in specific subcellular compartments, the ability to programme condensate localization provides a powerful means to modulate intracellular pathways. Another unique feature of RNA-based condensates is their ability to form discrete, multicomponent compartments solely through rationally designed KL interactions. This capability is difficult to achieve with synthetic protein-based condensates, which typically rely on intrinsically disordered regions that lack the base-pairing programmability of RNA^47^. Previously, recruitment of target RNAs into condensates relied on engineered recognition tags on the target RNA^48,49^. Here, we introduce a more general approach: direct sequence-specific hybridization between nanostars and target RNA sequences. This strategy eliminates the need for target RNA modification and may be applied to arbitrary transcripts, providing means to control native RNAs and pathways such as rRNA processing and mRNA translation^23,50,51^.

Overexpression of structured circular RNAs may contribute to metabolic stress, as suggested by our immunostaining results. This could be improved by inducible expression. Shell-like structures also warrant further investigation: they may originate from weak intermolecular interactions, such as G-quadruplex formation within fluorescent aptamers^52^, or from metabolic pathways associated with circular RNAs, as indicated by their interaction with G3BP1 (ref. ^26^). Alternatively, they could represent another form of condensate, akin to previously reported anisosomes^27^. With the growing recognition of RNA as a central player in cellular phase separation, we anticipate that future versions of this technology will achieve greater selectivity and adaptability, ultimately enabling the construction of synthetic organelles with tailored and novel biological functions.

## Methods

### Sequence design

Nanostars were designed using NUPACK^53^ on the basis of published in vitro results^11^. For each design, ten NUPACK trials were run, and the one that generated the lowest defect score was selected. Broccoli, Pepper, Mango and MS2 aptamer sequences were taken from literature^18^^,20^^,34^^,54^. The wild-type KL (5′-GCGCGC) was adapted from the HIV-1 palindromic KL sequence^32^. Orthogonal KLs 5′-UCGCGA, 5′-GUCGAC and 5′-GGUACC were taken from the study by Fabrini et al.^12^. KLs 5′-GUAUAC and 5′-UAUAUA were designed by simply replacing GC pairs with AU pairs. Non-palindromic KLs were adapted from the 3sβ set designed by Stewart et al.^11^. Detailed design principles of nanostar stems and KLs can be found in Supplementary Section 1.1. All sequences are listed in Supplementary Table 1.

### RNA synthesis for in vitro characterization

All RNA strands for in vitro experiments were transcribed from custom DNA templates synthesized by Integrated DNA Technologies as LabReady resuspensions with standard desalt purification. We annealed non-coding DNA templates with a 21-nt complement including the T7 promoter region and a 4-nt sealing domain (5′-GCGC). These templates were annealed in 1× TE/50 mM NaCl from 90 °C to room temperature at −1 °C min^−1^ at 5 μM for storage and used at 0.01 μM during in vitro transcription. RNA strands were transcribed in vitro at 37 °C using 7.5% (v/v) T7 polymerase from the AmpliScribe T7-Flash transcription kit (ASF3507, Biosearch Technologies), and transcription buffer prepared in-house: 40 mM of Tris-HCl, 10 mM of NaCl, 30 mM MgCl_2_, 2 mM spermidine, 7.5 mM of each NTP and 10 mM DTT.

### Plasmid development

Inserts were directly purchased from Integrated DNA Technologies as two single-stranded, 5′-phosphorylated oligonucleotides containing the sequence of interest, flanked by NotI and SacII restriction sites. The two strands were annealed in 50 mM NaCl and 1× TE buffer using a heat treatment protocol including a 5-min melt at 90 °C, followed by a slow temperature ramp at −1 °C min^−1^, and held at 20 °C. The resulting products were double-stranded DNA fragments with sticky ends ready for ligation. After annealing, strands were purified with a DNA cleanup kit (NEB, T1030). The DNA encoding the nanostar sequences was inserted in the pAV-U6+27-Tornado-Broccoli (Addgene, 261587) plasmid. Plasmids were prepared by (1) digestion with NotI-HF (NEB, R3189S) (2 μl for 20-μl reactions) at 37 °C for 1 h, (2) purification with the DNA cleanup kit, (3) digestion with SacII (NEB, R0157S) (2 μl for 20-μl reactions) at 37 °C for 1 h and (4) purification with a 0.8% 1× TAE agarose gel to select the product with the correct size. Digested backbones were finally purified using a gel extraction kit (Qiagen, 28704). Digested backbone and inserts were ligated at a 1:10 molecular ratio by overnight incubation with T4 DNA ligase (NEB, M0202S) at 4 °C. Ligated plasmids were transformed into 50 μl DF5Hα competent cells (Thermo Fisher, EC0112 and 18258012) following the manufacturer’s protocol. We then extracted plasmid DNA using a Miniprep kit (Qiagen, 27106) following the manufacturer’s protocol. Extracted plasmids were finally sequenced by Eurofins Genomics (whole plasmid sequencing service). The plasmid expressing MCP-mCherry was purchased from Addgene (207668)^55^.

### Cell culture and maintenance

HEK293T (ATCC, CRL-3216), HeLa (ATCC, CCL-2) and U-2 OS (ATCC, HTB-96) cells were grown in Dulbecco modified Eagle’s medium (DMEM), high glucose, pyruvate (Thermo Fisher, 11995065) containing 10% fetal bovine serum and 100 U ml^−1^ penicillin–streptomycin (Thermo Fisher) and maintained at 37 °C with 5% CO_2_ in a humidified incubator. Cells used for imaging were cultured in µ-Slide 8-well high slides (Ibidi GmbH).

### Transfection

Seeding density was adapted across cell types to achieve ~70% confluence at transfection. Lipofectamine 2000 (Thermo Fisher, 11668019) was used for transfecting HEK293T cells. FuGene HD (Promega, E2311) was used for transfecting HeLa and U-2 OS cells as it demonstrated less cytotoxicity due to transfection. For experiments involving the expression of multiple nanostars, the total amount of plasmid DNA used in each experiment was kept constant, with an equal proportion of each nanostar variant.

### Total RNA extraction

Cells were transfected in 24-well plates, as described in Supplementary Section 1.5. We changed the medium 24 h after transfection, and collected cells 48 h after transfection. For collection, we aspirated media, washed with PBS and trypsinized the cells. After trypsinization, cells were resuspended in PBS, lysed and RNA was purified using the Monarch Total RNA Miniprep Kit (NEB, T2010S). RNA concentration was estimated using a Nanodrop 2000c by measuring absorption at 260 nm.

### Live-cell staining

The culture medium from overnight incubation was aspirated and replaced with fresh medium supplemented with two drops of NucBlue Live reagent (Hoechst 33342 nuclear dye, Thermo Fisher, R37605) per millilitre of media, along with the appropriate staining dyes according to experimental conditions. For conditions involving the Broccoli aptamer, we used 40 µM of DFHBI (Lucerna; 400-5 mg). For experiments involving the Pepper aptamer, we supplied 10 nM of HBC620 (MedChemExpress, HY-133520). Live cells were then incubated for at least 15 min at 37 °C before imaging. Cells were imaged in the presence of dyes.

### Fixed cell staining and immunostaining

Mouse anti-coilin (Cajal body colocalization) was purchased from Abcam (ab11822; 1:1,900, 1 μg ml^−1^). Mouse anti-SC35 (nuclear speckle colocalization) was purchased from Abcam (ab11826; 1:200, 5 μg ml^−1^). Mouse anti-fibrillarin (nucleolus colocalization) was purchased from Antibodies.com (A85370; 1:200). Mouse anti-G3BP1 (stress granules colocalization) was purchased from Thermo Fisher (66486-1-IG; 1:200, 5 μg ml^−1^). Mouse anti-DCP1A (P-body colocalization) was purchased from Novus Biological (H00055802-M06; 1:200).

Before fixation, cell culture media were removed and cells were rinsed with PBS (Thermo Fisher, 10010023). Cells were then fixed in a PBS buffer (Thermo Fisher, 14190144) containing 4% paraformaldehyde (Thermo Fisher, 043368.9M) for 10 min at room temperature, and washed with the PBS buffer three times, each for 5 min. Next, we permeabilized cells with 0.5% Triton X-100 (Sigma-Aldrich, 9002-93-1) in PBS buffer for 10 min and washed three times. For imaging condensates involving the Mango aptamer, we added PBS supplemented with NucBlue reagent (Thermo Fisher, R37605) and 200 nM of TO1-B (ABM, G955). Cells were incubated in the buffer for 15 min before imaging. For experiments involving immunostaining, cells were further blocked using 3% bovine serum albumin (BSA) (w/v; Sigma-Aldrich, 9048-46-8) in PBS buffer for 1 h, and washed three times. Then, cells were stained with corresponding primary antibodies diluted to the above-mentioned concentrations with 3% BSA in PBS buffer and incubated at 4 °C overnight. The next day, primary antibodies were removed and cells were washed three times before the addition of secondary antibodies (Thermo Fisher, A-21236; 1:1,000 in 3% BSA in PBS buffer). We incubated cells in secondary antibodies for 1 h before removing the buffer and washing them three times with PBS. For the final wash, PBS was supplemented with 40 μM DFHBI and NucBlue reagent. Cells were incubated in the buffer for 15 min before imaging.

### Microscopy

FRAP and fusion experiments were performed with epifluorescence imaging using a Nikon Eclipse TI-E inverted microscope and a 60× oil immersion objective. *z*-Stack confocal images were acquired using a Nikon Ti microscope equipped with an NL5+ camera. Images in Fig. 4d,k were captured using a Yokogawa CSU X1 spinning disk confocal on an inverted Zeiss stand. Hoechst (NucBlue staining) signals were detected in the UV channel (excitation 405 nm). Broccoli aptamer fluorescence was measured using the GFP channel (excitation 488 nm). Mango aptamer fluorescence was measured using the YFP channel (excitation 514 nm). Pepper aptamer, CY3 and mCherry fluorescence was detected using the RFP channel (excitation 561 nm). Finally, Alexa Fluor 647-labelled secondary antibody fluorescence was detected using the 647 nm channel (excitation 647 nm).

### Image processing

To provide a more comprehensive view of condensate signals across all planes, confocal micrographs in the manuscript figures are max-pixel-intensity *z*-projections, unless otherwise specified in the figure caption. Detailed data processing methods and schematics can be found in Supplementary Information, including condensate volume and number quantification, nucleus volume quantification, partition coefficient calculation for linked condensates (*J*_R_ and *J*_G_), partition coefficient calculation for peptides and small-molecule dye, and the PCC and Manders’ overlap coefficient (M1 and M2) calculation.

### FRAP

FRAP experiments were performed using a Nikon Eclipse TI-E inverted microscope with a temperature control unit. In vitro samples were loaded into a house-made chamber and sealed with epoxy (Gorilla, 5-min set) for imaging. For in vivo experiments, cells were stained and imaged in the Ibidi chamber described above. Condensates were bleached with a 488-nm laser for 200 ms. For in vitro samples, imaging was captured once before bleaching and every 5 s for 10 min after bleaching. For in vivo samples, imaging was captured once before bleaching and every 200 ms for 2 min after bleaching or every 1.5 s for 5 min after bleaching. Images were analysed by extracting time-dependent average intensities within the bleached area and unbleached area. Data processing and fitting details are described in Supplementary Information.

### Time-dependent coalescence analysis

For in vitro experiments, RNA strands were transcribed, labelled with 1% CY3-UTP, diluted ten times with transcription buffer and sealed in a chamber with epoxy, following the same protocol as FRAP experiments. Samples were imaged every 5 min for the first 4 h, then every 20 min until 10 h. We monitored condensate fusion events using a Nikon Eclipse TI-E inverted microscope with a temperature control unit. The temperature was maintained at 37 °C for all experiments.

For in vivo fusion experiments, we imaged cells (in media supplemented with 40 μM DFHBI and 2 drops ml^−1^ NucBlue) every 5 min for 60 min under the confocal microscope. The temperature was maintained at 37 °C. Fusion events were identified manually. Data processing was performed using a script in Python3, as described in our previous work^11^.

### Flow cytometry

Flow cytometry experiments were performed using a BD FACSAria flow cytometer. Cells were transfected in 24-well plates, as described in Supplementary Section 1.5. We changed the media 24 h after transfection, and collected cells 48 h after transfection. For collection, we aspirated media, washed with PBS and trypsinized the cells. After trypsinization, cells were resuspended in PBS supplemented with 10% fetal bovine serum and dyes and filtered through a 40-μm cell strainer (Fisher Scientific, cat. no. 22363547) for flow cytometry. Hoechst was detected using a laser with Ex. 405 nm and a 450/50-nm filter; DFHBI was detected using a laser with Ex. 488 nm and a 530/30-nm filter.

### RT–qPCR

Reverse transcription was carried out with equal amounts of RNA using the Protoscript II First Strand cDNA Synthesis Kit and random hexamers (New England Biolabs). Reverse-transcription quantitative PCR (RT–qPCR) was then performed using tenfold diluted cDNA and the Luna Universal qPCR Master Mix (New England Biolabs) in the CFX Real-Time PCR system (Bio-Rad), courtesy of the UCLA Virology Core. The qPCR conditions used were as previously described^56^^,57^. Target transcript levels were determined by normalizing the cycle threshold value of the target transcript to that of the housekeeping gene *RPS11* transcript. Fold change was calculated using this normalized value relative to Lipofectamine control expression levels. For RT–qPCR primers, see Supplementary Table 4.

### Polyacrylamide gel electrophoresis

Gel pre-mix was prepared by adding 42 g of urea to nanopure water, the mixture was then heated until the urea completely dissolved. This mixture was allowed to cool to room temperature, and then a 40% (v/v) 19:1 acrylamide:bis-acrylamide solution was added in the appropriate volume for the desired percentage (final volume of 100 ml). To start polymerization, 8 ml of pre-mix was added in appropriate ratios with TBE and nanopure water, ammonium persulfate and tetramethylethylenediamine. Gels were cast in 8 cm × 8 cm, 1-mm-thick disposable mini gel cassettes (Thermo Scientific, NC2010) and allowed to polymerize for 30 min before electrophoresis. After curing, the gel was pre-run in a 1× TBE buffer for 30 min. Wells were washed carefully to remove excessive urea. Samples and a low-range ssRNA ladder (NEB, N0364S) were prepared by mixing individual strands with denaturing RNA loading dye (NEB, B0363S), then heated at 70 °C for 10 min and immediately placed on ice. Owing to the low expression of exogenous RNA in mammalian cells, 5 μg of total RNA extraction was loaded into each well. Gels were run at room temperature at 100 V in 1× TBE unless otherwise noted. After electrophoresis, the gels were washed three times, each for 5 min, with nanopure water, then stained with DFHBI-1T staining buffer (10 μM DFHBI-1T, 40 mM HEPES, 100 mM KCl and 1 mM MgCl_2_) for 15 min. After staining, gels were imaged using the Bio-Rad Gel Imaging Systems. Then, gels were washed three times, each for 5 min, to remove the DFHBI-1T and stained in 1× SYBR Gold Nucleic Acid Gel Stain for 15 min and imaged again.

### Statistics and reproducibility

At least three independent biological replicates were tested for each condition. For microscopy imaging, at least three different fields of view were captured for each replicate. No data were excluded from the analyses.

## Online content

Any methods, additional references, Nature Portfolio reporting summaries, source data, extended data, supplementary information, acknowledgements, peer review information; details of author contributions and competing interests; and statements of data and code availability are available at 10.1038/s41565-026-02164-7.

## Supplementary information

Supplementary Information Detailed methods, Supplementary Notes 1 and 2, Figs. 1–46 and Tables 1–4.

Supplementary Dataset 1 Oligonucleotide sequences and supplementary source data.

## Source data

Source Data Fig. 1 FRAP, fusion and qPCR data shown in Fig. 1.

Source Data Fig. 2 Condensate volume and number quantification in Fig. 2.

Source Data Fig. 3 Condensate volume and number quantification, as well as CCDFs, shown in Fig. 3.

Source Data Fig. 4 Condensate volume and number quantification, as well as Pearson coefficients, shown in Fig. 4.

Source Data Fig. 5 Mixing index shown in Fig. 5.

Source Data Fig. 6 Pearson coefficients shown in Fig. 6.

## Data Availability

All the data that support the findings of this study are available within the article and its Supplementary Information. Additional data are available via Zenodo at 10.5281/zenodo.17823527 (ref. ^58^). Source data are provided with this paper.

## Code availability

The data analysis code used in this article is available via GitHub at https://github.com/FrancoLabUCLA/Li-2025-RNA-condensate.
