# Supplementary figures and images for: Programmable artificial RNA condensates in mammalian cells (part 1 of 3)
Source: Nat Nanotechnol. 2026 Apr 29;21(6):821–30. doi: 10.1038/s41565-026-02164-7 (PMC13293865; doi:10.1038/s41565-026-02164-7)

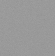

Supplement: Supplementary file 3 — FRAP, fusion and qPCR data shown in Fig. 1. [file 41565_2026_2164_MOESM3_ESM.zip › Source Data Fig. 1/Fusion (Figure 1 and SI Figure 15-17)/in vitro/ROI13.tif]

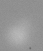

Supplement: Supplementary file 3 — FRAP, fusion and qPCR data shown in Fig. 1. [file 41565_2026_2164_MOESM3_ESM.zip › Source Data Fig. 1/Fusion (Figure 1 and SI Figure 15-17)/in vitro/ROI5.tif]

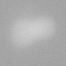

Supplement: Supplementary file 3 — FRAP, fusion and qPCR data shown in Fig. 1. [file 41565_2026_2164_MOESM3_ESM.zip › Source Data Fig. 1/Fusion (Figure 1 and SI Figure 15-17)/in vitro/ROI2.tif]

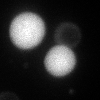

Supplement: Supplementary file 3 — FRAP, fusion and qPCR data shown in Fig. 1. [file 41565_2026_2164_MOESM3_ESM.zip › Source Data Fig. 1/Fusion (Figure 1 and SI Figure 15-17)/in vivo/MAX_20250530_JABr_Nuc40uMDFHBI_sample1_1_Pos0_100x100.tif]

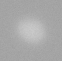

Supplement: Supplementary file 3 — FRAP, fusion and qPCR data shown in Fig. 1. [file 41565_2026_2164_MOESM3_ESM.zip › Source Data Fig. 1/Fusion (Figure 1 and SI Figure 15-17)/in vitro/ROI15.tif]

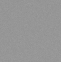

Supplement: Supplementary file 3 — FRAP, fusion and qPCR data shown in Fig. 1. [file 41565_2026_2164_MOESM3_ESM.zip › Source Data Fig. 1/Fusion (Figure 1 and SI Figure 15-17)/in vitro/ROI9.tif]

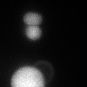

Supplement: Supplementary file 3 — FRAP, fusion and qPCR data shown in Fig. 1. [file 41565_2026_2164_MOESM3_ESM.zip › Source Data Fig. 1/Fusion (Figure 1 and SI Figure 15-17)/in vivo/MAX_20250530_JABr_Nuc40uMDFHBI_sample3_1_Pos2_87x87_cyto.tif]

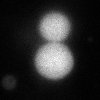

Supplement: Supplementary file 3 — FRAP, fusion and qPCR data shown in Fig. 1. [file 41565_2026_2164_MOESM3_ESM.zip › Source Data Fig. 1/Fusion (Figure 1 and SI Figure 15-17)/in vivo/midplane_20250530_JABr_Nuc40uMDFHBI_sample1_1_Pos4_100x100.tif]

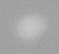

Supplement: Supplementary file 3 — FRAP, fusion and qPCR data shown in Fig. 1. [file 41565_2026_2164_MOESM3_ESM.zip › Source Data Fig. 1/Fusion (Figure 1 and SI Figure 15-17)/in vitro/ROI3.tif]

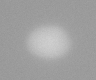

Supplement: Supplementary file 3 — FRAP, fusion and qPCR data shown in Fig. 1. [file 41565_2026_2164_MOESM3_ESM.zip › Source Data Fig. 1/Fusion (Figure 1 and SI Figure 15-17)/in vitro/ROI6.tif]

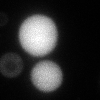

Supplement: Supplementary file 3 — FRAP, fusion and qPCR data shown in Fig. 1. [file 41565_2026_2164_MOESM3_ESM.zip › Source Data Fig. 1/Fusion (Figure 1 and SI Figure 15-17)/in vivo/MAX_20250530_JABr_Nuc40uMDFHBI_sample2_1_Pos3_100x100.tif]

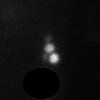

Supplement: Supplementary file 3 — FRAP, fusion and qPCR data shown in Fig. 1. [file 41565_2026_2164_MOESM3_ESM.zip › Source Data Fig. 1/Fusion (Figure 1 and SI Figure 15-17)/in vivo/20250530_JABr_Nuc40uMDFHBI_sample2_1_Pos3_cyto.tif]

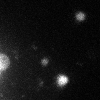

Supplement: Supplementary file 3 — FRAP, fusion and qPCR data shown in Fig. 1. [file 41565_2026_2164_MOESM3_ESM.zip › Source Data Fig. 1/Fusion (Figure 1 and SI Figure 15-17)/in vivo/MAX_20250530_JABr_Nuc40uMDFHBI_sample1_1_Pos2_100x100.tif]

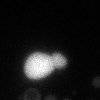

Supplement: Supplementary file 3 — FRAP, fusion and qPCR data shown in Fig. 1. [file 41565_2026_2164_MOESM3_ESM.zip › Source Data Fig. 1/Fusion (Figure 1 and SI Figure 15-17)/in vivo/MAX_20250530_JABr_Nuc40uMDFHBI_sample1_1_MMStack_Pos4_100x100_cyto.tif]

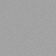

Supplement: Supplementary file 3 — FRAP, fusion and qPCR data shown in Fig. 1. [file 41565_2026_2164_MOESM3_ESM.zip › Source Data Fig. 1/Fusion (Figure 1 and SI Figure 15-17)/in vitro/ROI1.tif]

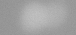

Supplement: Supplementary file 3 — FRAP, fusion and qPCR data shown in Fig. 1. [file 41565_2026_2164_MOESM3_ESM.zip › Source Data Fig. 1/Fusion (Figure 1 and SI Figure 15-17)/in vitro/ROI12.tif]

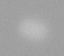

Supplement: Supplementary file 3 — FRAP, fusion and qPCR data shown in Fig. 1. [file 41565_2026_2164_MOESM3_ESM.zip › Source Data Fig. 1/Fusion (Figure 1 and SI Figure 15-17)/in vitro/ROI8.tif]

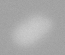

Supplement: Supplementary file 3 — FRAP, fusion and qPCR data shown in Fig. 1. [file 41565_2026_2164_MOESM3_ESM.zip › Source Data Fig. 1/Fusion (Figure 1 and SI Figure 15-17)/in vitro/ROI4.tif]

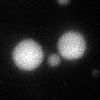

Supplement: Supplementary file 3 — FRAP, fusion and qPCR data shown in Fig. 1. [file 41565_2026_2164_MOESM3_ESM.zip › Source Data Fig. 1/Fusion (Figure 1 and SI Figure 15-17)/in vivo/MAX_20250530_JABr_Nuc40uMDFHBI_sample1_1_Pos2_100x100_cyto.tif]

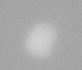

Supplement: Supplementary file 3 — FRAP, fusion and qPCR data shown in Fig. 1. [file 41565_2026_2164_MOESM3_ESM.zip › Source Data Fig. 1/Fusion (Figure 1 and SI Figure 15-17)/in vitro/ROI11.tif]

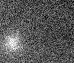

Supplement: Supplementary file 3 — FRAP, fusion and qPCR data shown in Fig. 1. [file 41565_2026_2164_MOESM3_ESM.zip › Source Data Fig. 1/Fusion (Figure 1 and SI Figure 15-17)/in vitro/ROI7.tif]

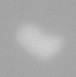

Supplement: Supplementary file 3 — FRAP, fusion and qPCR data shown in Fig. 1. [file 41565_2026_2164_MOESM3_ESM.zip › Source Data Fig. 1/Fusion (Figure 1 and SI Figure 15-17)/in vitro/ROI10.tif]

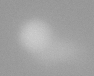

Supplement: Supplementary file 3 — FRAP, fusion and qPCR data shown in Fig. 1. [file 41565_2026_2164_MOESM3_ESM.zip › Source Data Fig. 1/Fusion (Figure 1 and SI Figure 15-17)/in vitro/ROI14.tif]

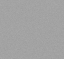

Supplement: Supplementary file 3 — FRAP, fusion and qPCR data shown in Fig. 1. [file 41565_2026_2164_MOESM3_ESM.zip › Source Data Fig. 1/Fusion (Figure 1 and SI Figure 15-17)/in vitro/ROI16.tif]

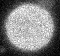

Supplement: Supplementary file 7 — Mixing index shown in Fig. 5. [file 41565_2026_2164_MOESM7_ESM.zip › Source Data Fig. 5/Mixing index (Figure 5)/1to1to1_4arm/raw/20240801_4arm_1to1to1_NucDFHBIHBC_20-40-50_Sample2_2_cell1_cond1.tif]

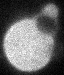

Supplement: Supplementary file 7 — Mixing index shown in Fig. 5. [file 41565_2026_2164_MOESM7_ESM.zip › Source Data Fig. 5/Mixing index (Figure 5)/1to1to1_4arm/raw/20240801_4arm_1to1to1_NucDFHBIHBC_20-40-50_Sample1_4_cell2_cond1.tif]

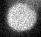

Supplement: Supplementary file 7 — Mixing index shown in Fig. 5. [file 41565_2026_2164_MOESM7_ESM.zip › Source Data Fig. 5/Mixing index (Figure 5)/1to1to1_4arm/raw/20240801_4arm_1to1to1_NucDFHBIHBC_20-40-50_Sample3_1_cell1_cond2.tif]

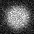

Supplement: Supplementary file 7 — Mixing index shown in Fig. 5. [file 41565_2026_2164_MOESM7_ESM.zip › Source Data Fig. 5/Mixing index (Figure 5)/1to1to1_4arm/raw/20240801_4arm_1to1to1_NucDFHBIHBC_20-40-50_Sample3_3_cell2_cond2.tif]

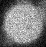

Supplement: Supplementary file 7 — Mixing index shown in Fig. 5. [file 41565_2026_2164_MOESM7_ESM.zip › Source Data Fig. 5/Mixing index (Figure 5)/1to1to1_4arm/raw/20240801_4arm_1to1to1_NucDFHBIHBC_20-40-50_Sample1_4_cell1_cond2.tif]

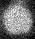

Supplement: Supplementary file 7 — Mixing index shown in Fig. 5. [file 41565_2026_2164_MOESM7_ESM.zip › Source Data Fig. 5/Mixing index (Figure 5)/1to1to1_4arm/raw/20240801_4arm_1to1to1_NucDFHBIHBC_20-40-50_Sample3_3_cell1_cond1.tif]

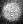

Supplement: Supplementary file 7 — Mixing index shown in Fig. 5. [file 41565_2026_2164_MOESM7_ESM.zip › Source Data Fig. 5/Mixing index (Figure 5)/1to1to1_4arm/raw/20240801_4arm_1to1to1_NucDFHBIHBC_20-40-50_Sample2_1_cell1_cond1.tif]

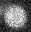

Supplement: Supplementary file 7 — Mixing index shown in Fig. 5. [file 41565_2026_2164_MOESM7_ESM.zip › Source Data Fig. 5/Mixing index (Figure 5)/1to1to1_4arm/raw/20240801_4arm_1to1to1_NucDFHBIHBC_20-40-50_Sample3_2_cell1_cond3.tif]

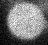

Supplement: Supplementary file 7 — Mixing index shown in Fig. 5. [file 41565_2026_2164_MOESM7_ESM.zip › Source Data Fig. 5/Mixing index (Figure 5)/1to1to1_4arm/raw/20240801_4arm_1to1to1_NucDFHBIHBC_20-40-50_Sample2_1_cell1_cond3.tif]

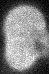

Supplement: Supplementary file 7 — Mixing index shown in Fig. 5. [file 41565_2026_2164_MOESM7_ESM.zip › Source Data Fig. 5/Mixing index (Figure 5)/1to1to1_4arm/raw/20240801_4arm_1to1to1_NucDFHBIHBC_20-40-50_Sample2_2_cell1_cond2.tif]

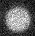

Supplement: Supplementary file 7 — Mixing index shown in Fig. 5. [file 41565_2026_2164_MOESM7_ESM.zip › Source Data Fig. 5/Mixing index (Figure 5)/1to1to1_4arm/raw/20240801_4arm_1to1to1_NucDFHBIHBC_20-40-50_Sample2_3_cell1_cond2.tif]

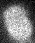

Supplement: Supplementary file 7 — Mixing index shown in Fig. 5. [file 41565_2026_2164_MOESM7_ESM.zip › Source Data Fig. 5/Mixing index (Figure 5)/1to1to1_4arm/raw/20240801_4arm_1to1to1_NucDFHBIHBC_20-40-50_Sample2_1_cell1_cond2.tif]

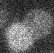

Supplement: Supplementary file 7 — Mixing index shown in Fig. 5. [file 41565_2026_2164_MOESM7_ESM.zip › Source Data Fig. 5/Mixing index (Figure 5)/1to1to1_4arm/raw/20240801_4arm_1to1to1_NucDFHBIHBC_20-40-50_Sample3_3_cell1_cond2.tif]

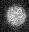

Supplement: Supplementary file 7 — Mixing index shown in Fig. 5. [file 41565_2026_2164_MOESM7_ESM.zip › Source Data Fig. 5/Mixing index (Figure 5)/1to1to1_4arm/raw/20240801_4arm_1to1to1_NucDFHBIHBC_20-40-50_Sample3_3_cell2_cond3.tif]

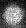

Supplement: Supplementary file 7 — Mixing index shown in Fig. 5. [file 41565_2026_2164_MOESM7_ESM.zip › Source Data Fig. 5/Mixing index (Figure 5)/1to1to1_4arm/raw/20240801_4arm_1to1to1_NucDFHBIHBC_20-40-50_Sample2_1_cell1_cond4.tif]

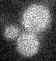

Supplement: Supplementary file 7 — Mixing index shown in Fig. 5. [file 41565_2026_2164_MOESM7_ESM.zip › Source Data Fig. 5/Mixing index (Figure 5)/1to1to1_4arm/raw/20240801_4arm_1to1to1_NucDFHBIHBC_20-40-50_Sample3_2_cell1_cond2.tif]

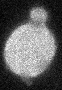

Supplement: Supplementary file 7 — Mixing index shown in Fig. 5. [file 41565_2026_2164_MOESM7_ESM.zip › Source Data Fig. 5/Mixing index (Figure 5)/1to1to1_4arm/raw/20240801_4arm_1to1to1_NucDFHBIHBC_20-40-50_Sample1_5_cell1_cond1.tif]

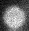

Supplement: Supplementary file 7 — Mixing index shown in Fig. 5. [file 41565_2026_2164_MOESM7_ESM.zip › Source Data Fig. 5/Mixing index (Figure 5)/1to1to1_4arm/raw/20240801_4arm_1to1to1_NucDFHBIHBC_20-40-50_Sample3_1_cell1_cond3.tif]

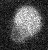

Supplement: Supplementary file 7 — Mixing index shown in Fig. 5. [file 41565_2026_2164_MOESM7_ESM.zip › Source Data Fig. 5/Mixing index (Figure 5)/1to1to1_4arm/raw/20240801_4arm_1to1to1_NucDFHBIHBC_20-40-50_Sample2_3_cell1_cond1.tif]

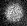

Supplement: Supplementary file 7 — Mixing index shown in Fig. 5. [file 41565_2026_2164_MOESM7_ESM.zip › Source Data Fig. 5/Mixing index (Figure 5)/1to1to1_4arm/raw/20240801_4arm_1to1to1_NucDFHBIHBC_20-40-50_Sample3_3_cell2_cond1.tif]

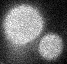

Supplement: Supplementary file 7 — Mixing index shown in Fig. 5. [file 41565_2026_2164_MOESM7_ESM.zip › Source Data Fig. 5/Mixing index (Figure 5)/1to1to1_4arm/raw/20240801_4arm_1to1to1_NucDFHBIHBC_20-40-50_Sample2_2_cell1_cond3.tif]

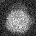

Supplement: Supplementary file 7 — Mixing index shown in Fig. 5. [file 41565_2026_2164_MOESM7_ESM.zip › Source Data Fig. 5/Mixing index (Figure 5)/1to1to1_4arm/raw/20240801_4arm_1to1to1_NucDFHBIHBC_20-40-50_Sample3_3_cell1_cond3.tif]

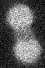

Supplement: Supplementary file 7 — Mixing index shown in Fig. 5. [file 41565_2026_2164_MOESM7_ESM.zip › Source Data Fig. 5/Mixing index (Figure 5)/1to1to1_4arm/raw/20240801_4arm_1to1to1_NucDFHBIHBC_20-40-50_Sample2_3_cell1_cond3.tif]

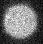

Supplement: Supplementary file 7 — Mixing index shown in Fig. 5. [file 41565_2026_2164_MOESM7_ESM.zip › Source Data Fig. 5/Mixing index (Figure 5)/1to1to1_4arm/raw/20240801_4arm_1to1to1_NucDFHBIHBC_20-40-50_Sample3_2_cell1_cond1.tif]

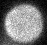

Supplement: Supplementary file 7 — Mixing index shown in Fig. 5. [file 41565_2026_2164_MOESM7_ESM.zip › Source Data Fig. 5/Mixing index (Figure 5)/1to1to1_4arm/raw/20240801_4arm_1to1to1_NucDFHBIHBC_20-40-50_Sample3_1_cell1_cond1.tif]

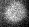

Supplement: Supplementary file 7 — Mixing index shown in Fig. 5. [file 41565_2026_2164_MOESM7_ESM.zip › Source Data Fig. 5/Mixing index (Figure 5)/1to1to1_4arm/raw/20240801_4arm_1to1to1_NucDFHBIHBC_20-40-50_Sample2_1_cell1_cond5.tif]

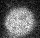

Supplement: Supplementary file 7 — Mixing index shown in Fig. 5. [file 41565_2026_2164_MOESM7_ESM.zip › Source Data Fig. 5/Mixing index (Figure 5)/1to1to1_4arm/raw/20240801_4arm_1to1to1_NucDFHBIHBC_20-40-50_Sample1_4_cell1_cond1.tif]

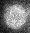

Supplement: Supplementary file 7 — Mixing index shown in Fig. 5. [file 41565_2026_2164_MOESM7_ESM.zip › Source Data Fig. 5/Mixing index (Figure 5)/1to1to1_4arm/raw/20240801_4arm_1to1to1_NucDFHBIHBC_20-40-50_Sample1_3_cell3_cond3.tif]

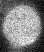

Supplement: Supplementary file 7 — Mixing index shown in Fig. 5. [file 41565_2026_2164_MOESM7_ESM.zip › Source Data Fig. 5/Mixing index (Figure 5)/1to1to1_4arm/raw/20240801_4arm_1to1to1_NucDFHBIHBC_20-40-50_Sample1_3_cell3_cond1.tif]

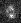

Supplement: Supplementary file 7 — Mixing index shown in Fig. 5. [file 41565_2026_2164_MOESM7_ESM.zip › Source Data Fig. 5/Mixing index (Figure 5)/2to1to2_2arm/raw/20240411_Astem_2armlinker_212_40uMDFHBI_10nMHBC620_20-40-50_Sample1_1_cell1_cond3.tif]

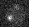

Supplement: Supplementary file 7 — Mixing index shown in Fig. 5. [file 41565_2026_2164_MOESM7_ESM.zip › Source Data Fig. 5/Mixing index (Figure 5)/2to1to2_2arm/raw/20240411_Astem_2armlinker_212_40uMDFHBI_10nMHBC620_20-40-50_Sample2_3_cell3_cond1.tif]

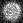

Supplement: Supplementary file 7 — Mixing index shown in Fig. 5. [file 41565_2026_2164_MOESM7_ESM.zip › Source Data Fig. 5/Mixing index (Figure 5)/1to3to1_2arm/raw/20240425_1to3to1_linker_2arm_sample3_40uMDFHBI_10nMHBC620_20_40_50_3_cell1_cond4.tif]

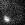

Supplement: Supplementary file 7 — Mixing index shown in Fig. 5. [file 41565_2026_2164_MOESM7_ESM.zip › Source Data Fig. 5/Mixing index (Figure 5)/2to1to2_2arm/raw/20240411_Astem_2armlinker_212_40uMDFHBI_10nMHBC620_20-40-50_Sample2_3_cell2_cond5.tif]

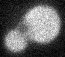

Supplement: Supplementary file 7 — Mixing index shown in Fig. 5. [file 41565_2026_2164_MOESM7_ESM.zip › Source Data Fig. 5/Mixing index (Figure 5)/1to1to1_4arm/raw/20240801_4arm_1to1to1_NucDFHBIHBC_20-40-50_Sample1_3_cell2_cond1.tif]

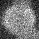

Supplement: Supplementary file 7 — Mixing index shown in Fig. 5. [file 41565_2026_2164_MOESM7_ESM.zip › Source Data Fig. 5/Mixing index (Figure 5)/1to3to1_2arm/raw/20240425_1to3to1_linker_2arm_sample2_40uMDFHBI_10nMHBC620_20_40_50_2_cell3_cond1.tif]

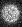

Supplement: Supplementary file 7 — Mixing index shown in Fig. 5. [file 41565_2026_2164_MOESM7_ESM.zip › Source Data Fig. 5/Mixing index (Figure 5)/1to3to1_2arm/raw/20240425_1to3to1_linker_2arm_sample3_40uMDFHBI_10nMHBC620_20_40_50_3_cell1_cond1.tif]

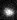

Supplement: Supplementary file 7 — Mixing index shown in Fig. 5. [file 41565_2026_2164_MOESM7_ESM.zip › Source Data Fig. 5/Mixing index (Figure 5)/2to1to2_2arm/raw/20240411_Astem_2armlinker_212_40uMDFHBI_10nMHBC620_20-40-50_Sample2_1_cell1_cond2.tif]

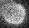

Supplement: Supplementary file 7 — Mixing index shown in Fig. 5. [file 41565_2026_2164_MOESM7_ESM.zip › Source Data Fig. 5/Mixing index (Figure 5)/1to3to1_2arm/raw/20240425_1to3to1_linker_2arm_sample2_40uMDFHBI_10nMHBC620_20_40_50_3_cell2_cond2.tif]

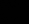

Supplement: Supplementary file 7 — Mixing index shown in Fig. 5. [file 41565_2026_2164_MOESM7_ESM.zip › Source Data Fig. 5/Mixing index (Figure 5)/2to1to2_4arm/raw/20240411_Astem_4armlinker_212_40uMDFHBI_10nMHBC620_20-40-50_Sample1_2_cell1_cond3.tif]

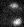

Supplement: Supplementary file 7 — Mixing index shown in Fig. 5. [file 41565_2026_2164_MOESM7_ESM.zip › Source Data Fig. 5/Mixing index (Figure 5)/2to1to2_2arm/raw/20240411_Astem_2armlinker_212_40uMDFHBI_10nMHBC620_20-40-50_Sample2_3_cell2_cond4.tif]

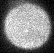

Supplement: Supplementary file 7 — Mixing index shown in Fig. 5. [file 41565_2026_2164_MOESM7_ESM.zip › Source Data Fig. 5/Mixing index (Figure 5)/1to1to1_4arm/raw/20240801_4arm_1to1to1_NucDFHBIHBC_20-40-50_Sample1_3_cell1_cond1.tif]

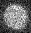

Supplement: Supplementary file 7 — Mixing index shown in Fig. 5. [file 41565_2026_2164_MOESM7_ESM.zip › Source Data Fig. 5/Mixing index (Figure 5)/1to3to1_2arm/raw/20240425_1to3to1_linker_2arm_sample2_40uMDFHBI_10nMHBC620_20_40_50_2_cell3_cond2.tif]

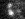

Supplement: Supplementary file 7 — Mixing index shown in Fig. 5. [file 41565_2026_2164_MOESM7_ESM.zip › Source Data Fig. 5/Mixing index (Figure 5)/2to1to2_2arm/raw/20240411_Astem_2armlinker_212_40uMDFHBI_10nMHBC620_20-40-50_Sample1_1_cell1_cond1.tif]

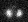

Supplement: Supplementary file 7 — Mixing index shown in Fig. 5. [file 41565_2026_2164_MOESM7_ESM.zip › Source Data Fig. 5/Mixing index (Figure 5)/2to1to2_2arm/raw/20240411_Astem_2armlinker_212_40uMDFHBI_10nMHBC620_20-40-50_Sample1_4_cell1_cond3.tif]

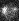

Supplement: Supplementary file 7 — Mixing index shown in Fig. 5. [file 41565_2026_2164_MOESM7_ESM.zip › Source Data Fig. 5/Mixing index (Figure 5)/2to1to2_2arm/raw/20240411_Astem_2armlinker_212_40uMDFHBI_10nMHBC620_20-40-50_Sample1_1_cell1_cond2.tif]

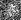

Supplement: Supplementary file 7 — Mixing index shown in Fig. 5. [file 41565_2026_2164_MOESM7_ESM.zip › Source Data Fig. 5/Mixing index (Figure 5)/2to1to2_4arm/raw/20240411_Astem_4armlinker_212_40uMDFHBI_10nMHBC620_20-40-50_Sample1_2_cell1_cond1.tif]

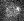

Supplement: Supplementary file 7 — Mixing index shown in Fig. 5. [file 41565_2026_2164_MOESM7_ESM.zip › Source Data Fig. 5/Mixing index (Figure 5)/2to1to2_2arm/raw/20240411_Astem_2armlinker_212_40uMDFHBI_10nMHBC620_20-40-50_Sample1_1_cell1_cond4.tif]

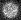

Supplement: Supplementary file 7 — Mixing index shown in Fig. 5. [file 41565_2026_2164_MOESM7_ESM.zip › Source Data Fig. 5/Mixing index (Figure 5)/1to3to1_2arm/raw/20240425_1to3to1_linker_2arm_sample3_40uMDFHBI_10nMHBC620_20_40_50_3_cell1_cond3.tif]

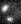

Supplement: Supplementary file 7 — Mixing index shown in Fig. 5. [file 41565_2026_2164_MOESM7_ESM.zip › Source Data Fig. 5/Mixing index (Figure 5)/2to1to2_2arm/raw/20240411_Astem_2armlinker_212_40uMDFHBI_10nMHBC620_20-40-50_Sample2_3_cell1_cond4.tif]

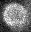

Supplement: Supplementary file 7 — Mixing index shown in Fig. 5. [file 41565_2026_2164_MOESM7_ESM.zip › Source Data Fig. 5/Mixing index (Figure 5)/1to3to1_2arm/raw/20240425_1to3to1_linker_2arm_sample2_40uMDFHBI_10nMHBC620_20_40_50_3_cell2_cond1.tif]

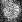

Supplement: Supplementary file 7 — Mixing index shown in Fig. 5. [file 41565_2026_2164_MOESM7_ESM.zip › Source Data Fig. 5/Mixing index (Figure 5)/1to3to1_2arm/raw/20240425_1to3to1_linker_2arm_sample2_40uMDFHBI_10nMHBC620_20_40_50_3_cell1_cond3.tif]

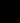

Supplement: Supplementary file 7 — Mixing index shown in Fig. 5. [file 41565_2026_2164_MOESM7_ESM.zip › Source Data Fig. 5/Mixing index (Figure 5)/2to1to2_4arm/raw/20240411_Astem_4armlinker_212_40uMDFHBI_10nMHBC620_20-40-50_Sample1_2_cell1_cond2.tif]

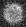

Supplement: Supplementary file 7 — Mixing index shown in Fig. 5. [file 41565_2026_2164_MOESM7_ESM.zip › Source Data Fig. 5/Mixing index (Figure 5)/1to3to1_2arm/raw/20240425_1to3to1_linker_2arm_sample2_40uMDFHBI_10nMHBC620_20_40_50_3_cell1_cond2.tif]

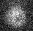

Supplement: Supplementary file 7 — Mixing index shown in Fig. 5. [file 41565_2026_2164_MOESM7_ESM.zip › Source Data Fig. 5/Mixing index (Figure 5)/2to1to2_4arm/raw/20240411_Astem_4armlinker_212_40uMDFHBI_10nMHBC620_20-40-50_Sample1_1_cell1_cond1.tif]

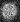

Supplement: Supplementary file 7 — Mixing index shown in Fig. 5. [file 41565_2026_2164_MOESM7_ESM.zip › Source Data Fig. 5/Mixing index (Figure 5)/1to3to1_2arm/raw/20240425_1to3to1_linker_2arm_sample3_40uMDFHBI_10nMHBC620_20_40_50_3_cell1_cond2.tif]

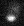

Supplement: Supplementary file 7 — Mixing index shown in Fig. 5. [file 41565_2026_2164_MOESM7_ESM.zip › Source Data Fig. 5/Mixing index (Figure 5)/2to1to2_2arm/raw/20240411_Astem_2armlinker_212_40uMDFHBI_10nMHBC620_20-40-50_Sample2_1_cell1_cond1.tif]

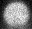

Supplement: Supplementary file 7 — Mixing index shown in Fig. 5. [file 41565_2026_2164_MOESM7_ESM.zip › Source Data Fig. 5/Mixing index (Figure 5)/1to1to1_4arm/raw/20240801_4arm_1to1to1_NucDFHBIHBC_20-40-50_Sample1_3_cell1_cond2.tif]

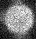

Supplement: Supplementary file 7 — Mixing index shown in Fig. 5. [file 41565_2026_2164_MOESM7_ESM.zip › Source Data Fig. 5/Mixing index (Figure 5)/1to3to1_2arm/raw/20240425_1to3to1_linker_2arm_sample2_40uMDFHBI_10nMHBC620_20_40_50_2_cell2_cond2.tif]

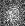

Supplement: Supplementary file 7 — Mixing index shown in Fig. 5. [file 41565_2026_2164_MOESM7_ESM.zip › Source Data Fig. 5/Mixing index (Figure 5)/2to1to2_4arm/raw/20240411_Astem_4armlinker_212_40uMDFHBI_10nMHBC620_20-40-50_Sample1_3_cell1_cond2.tif]

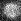

Supplement: Supplementary file 7 — Mixing index shown in Fig. 5. [file 41565_2026_2164_MOESM7_ESM.zip › Source Data Fig. 5/Mixing index (Figure 5)/1to3to1_2arm/raw/20240425_1to3to1_linker_2arm_sample2_40uMDFHBI_10nMHBC620_20_40_50_3_cell1_cond1.tif]

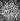

Supplement: Supplementary file 7 — Mixing index shown in Fig. 5. [file 41565_2026_2164_MOESM7_ESM.zip › Source Data Fig. 5/Mixing index (Figure 5)/2to1to2_4arm/raw/20240411_Astem_4armlinker_212_40uMDFHBI_10nMHBC620_20-40-50_Sample1_3_cell1_cond1.tif]

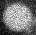

Supplement: Supplementary file 7 — Mixing index shown in Fig. 5. [file 41565_2026_2164_MOESM7_ESM.zip › Source Data Fig. 5/Mixing index (Figure 5)/1to1to1_4arm/raw/20240801_4arm_1to1to1_NucDFHBIHBC_20-40-50_Sample1_3_cell3_cond2.tif]

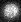

Supplement: Supplementary file 7 — Mixing index shown in Fig. 5. [file 41565_2026_2164_MOESM7_ESM.zip › Source Data Fig. 5/Mixing index (Figure 5)/1to3to1_2arm/raw/20240425_1to3to1_linker_2arm_sample2_40uMDFHBI_10nMHBC620_20_40_50_2_cell2_cond3.tif]

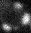

Supplement: Supplementary file 7 — Mixing index shown in Fig. 5. [file 41565_2026_2164_MOESM7_ESM.zip › Source Data Fig. 5/Mixing index (Figure 5)/2to1to2_2arm/raw/20240411_Astem_2armlinker_212_40uMDFHBI_10nMHBC620_20-40-50_Sample1_4_cell1_cond1.tif]

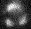

Supplement: Supplementary file 7 — Mixing index shown in Fig. 5. [file 41565_2026_2164_MOESM7_ESM.zip › Source Data Fig. 5/Mixing index (Figure 5)/2to1to2_2arm/raw/20240411_Astem_2armlinker_212_40uMDFHBI_10nMHBC620_20-40-50_Sample1_4_cell1_cond2.tif]

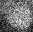

Supplement: Supplementary file 7 — Mixing index shown in Fig. 5. [file 41565_2026_2164_MOESM7_ESM.zip › Source Data Fig. 5/Mixing index (Figure 5)/2to1to2_4arm/raw/20240411_Astem_4armlinker_212_40uMDFHBI_10nMHBC620_20-40-50_Sample3_2_cell1_cond2.tif]

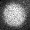

Supplement: Supplementary file 7 — Mixing index shown in Fig. 5. [file 41565_2026_2164_MOESM7_ESM.zip › Source Data Fig. 5/Mixing index (Figure 5)/1to3to1_2arm/raw/20240425_1to3to1_linker_2arm_sample2_40uMDFHBI_10nMHBC620_20_40_50_2_cell1_cond2.tif]

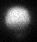

Supplement: Supplementary file 7 — Mixing index shown in Fig. 5. [file 41565_2026_2164_MOESM7_ESM.zip › Source Data Fig. 5/Mixing index (Figure 5)/2to1to2_2arm/raw/20240411_Astem_2armlinker_212_40uMDFHBI_10nMHBC620_20-40-50_Sample3_1_cell1_cond2.tif]

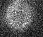

Supplement: Supplementary file 7 — Mixing index shown in Fig. 5. [file 41565_2026_2164_MOESM7_ESM.zip › Source Data Fig. 5/Mixing index (Figure 5)/2to1to2_4arm/raw/20240411_Astem_4armlinker_212_40uMDFHBI_10nMHBC620_20-40-50_Sample2_1_cell1_cond1.tif]

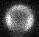

Supplement: Supplementary file 7 — Mixing index shown in Fig. 5. [file 41565_2026_2164_MOESM7_ESM.zip › Source Data Fig. 5/Mixing index (Figure 5)/1to3to1_2arm/raw/20240425_1to3to1_linker_2arm_sample3_40uMDFHBI_10nMHBC620_20_40_50_2_cell1_cond3.tif]

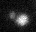

Supplement: Supplementary file 7 — Mixing index shown in Fig. 5. [file 41565_2026_2164_MOESM7_ESM.zip › Source Data Fig. 5/Mixing index (Figure 5)/2to1to2_2arm/raw/20240411_Astem_2armlinker_212_40uMDFHBI_10nMHBC620_20-40-50_Sample1_4_cell1_cond6.tif]

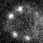

Supplement: Supplementary file 7 — Mixing index shown in Fig. 5. [file 41565_2026_2164_MOESM7_ESM.zip › Source Data Fig. 5/Mixing index (Figure 5)/2to1to2_2arm/raw/20240411_Astem_2armlinker_212_40uMDFHBI_10nMHBC620_20-40-50_Sample1_4_cell1_cond4.tif]

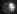

Supplement: Supplementary file 7 — Mixing index shown in Fig. 5. [file 41565_2026_2164_MOESM7_ESM.zip › Source Data Fig. 5/Mixing index (Figure 5)/1to2to1_2arm/raw/20240411_Astem_2armlinker_121_40uMDFHBI_10nMHBC620_20-40-50_Sample3_4_cell1_cond3.tif]

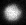

Supplement: Supplementary file 7 — Mixing index shown in Fig. 5. [file 41565_2026_2164_MOESM7_ESM.zip › Source Data Fig. 5/Mixing index (Figure 5)/1to2to1_2arm/raw/20240411_Astem_2armlinker_121_40uMDFHBI_10nMHBC620_20-40-50_Sample3_4_cell2_cond1.tif]

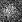

Supplement: Supplementary file 7 — Mixing index shown in Fig. 5. [file 41565_2026_2164_MOESM7_ESM.zip › Source Data Fig. 5/Mixing index (Figure 5)/2to1to2_4arm/raw/20240411_Astem_4armlinker_212_40uMDFHBI_10nMHBC620_20-40-50_Sample1_3_cell1_cond3.tif]

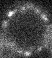

Supplement: Supplementary file 7 — Mixing index shown in Fig. 5. [file 41565_2026_2164_MOESM7_ESM.zip › Source Data Fig. 5/Mixing index (Figure 5)/2to1to2_2arm/raw/20240411_Astem_2armlinker_212_40uMDFHBI_10nMHBC620_20-40-50_Sample1_4_cell1_cond5.tif]
