# Supplementary figures and images for: Programmable artificial RNA condensates in mammalian cells (part 2 of 3)
Source: Nat Nanotechnol. 2026 Apr 29;21(6):821–30. doi: 10.1038/s41565-026-02164-7 (PMC13293865; doi:10.1038/s41565-026-02164-7)

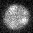

Supplement: Supplementary file 7 — Mixing index shown in Fig. 5. [file 41565_2026_2164_MOESM7_ESM.zip › Source Data Fig. 5/Mixing index (Figure 5)/2to1to2_4arm/raw/20240411_Astem_4armlinker_212_40uMDFHBI_10nMHBC620_20-40-50_Sample2_2_cell2_cond2.tif]

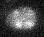

Supplement: Supplementary file 7 — Mixing index shown in Fig. 5. [file 41565_2026_2164_MOESM7_ESM.zip › Source Data Fig. 5/Mixing index (Figure 5)/2to1to2_4arm/raw/20240411_Astem_4armlinker_212_40uMDFHBI_10nMHBC620_20-40-50_Sample2_2_cell2_cond1.tif]

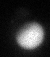

Supplement: Supplementary file 7 — Mixing index shown in Fig. 5. [file 41565_2026_2164_MOESM7_ESM.zip › Source Data Fig. 5/Mixing index (Figure 5)/2to1to2_2arm/raw/20240411_Astem_2armlinker_212_40uMDFHBI_10nMHBC620_20-40-50_Sample3_1_cell1_cond1.tif]

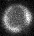

Supplement: Supplementary file 7 — Mixing index shown in Fig. 5. [file 41565_2026_2164_MOESM7_ESM.zip › Source Data Fig. 5/Mixing index (Figure 5)/1to3to1_2arm/raw/20240425_1to3to1_linker_2arm_sample3_40uMDFHBI_10nMHBC620_20_40_50_2_cell1_cond4.tif]

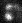

Supplement: Supplementary file 7 — Mixing index shown in Fig. 5. [file 41565_2026_2164_MOESM7_ESM.zip › Source Data Fig. 5/Mixing index (Figure 5)/2to1to2_2arm/raw/20240411_Astem_2armlinker_212_40uMDFHBI_10nMHBC620_20-40-50_Sample3_3_cell2_cond2.tif]

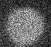

Supplement: Supplementary file 7 — Mixing index shown in Fig. 5. [file 41565_2026_2164_MOESM7_ESM.zip › Source Data Fig. 5/Mixing index (Figure 5)/2to1to2_4arm/raw/20240411_Astem_4armlinker_212_40uMDFHBI_10nMHBC620_20-40-50_Sample2_1_cell1_cond2.tif]

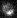

Supplement: Supplementary file 7 — Mixing index shown in Fig. 5. [file 41565_2026_2164_MOESM7_ESM.zip › Source Data Fig. 5/Mixing index (Figure 5)/1to2to1_2arm/raw/20240411_Astem_2armlinker_121_40uMDFHBI_10nMHBC620_20-40-50_Sample3_4_cell1_cond1.tif]

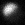

Supplement: Supplementary file 7 — Mixing index shown in Fig. 5. [file 41565_2026_2164_MOESM7_ESM.zip › Source Data Fig. 5/Mixing index (Figure 5)/1to2to1_2arm/raw/20240411_Astem_2armlinker_121_40uMDFHBI_10nMHBC620_20-40-50_Sample2_4_cell1_cond2.tif]

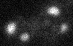

Supplement: Supplementary file 7 — Mixing index shown in Fig. 5. [file 41565_2026_2164_MOESM7_ESM.zip › Source Data Fig. 5/Mixing index (Figure 5)/2to1to2_2arm/raw/20240411_Astem_2armlinker_212_40uMDFHBI_10nMHBC620_20-40-50_Sample3_3_cell1_cond1.tif]

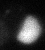

Supplement: Supplementary file 7 — Mixing index shown in Fig. 5. [file 41565_2026_2164_MOESM7_ESM.zip › Source Data Fig. 5/Mixing index (Figure 5)/2to1to2_2arm/raw/20240411_Astem_2armlinker_212_40uMDFHBI_10nMHBC620_20-40-50_Sample3_1_cell1_cond3.tif]

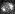

Supplement: Supplementary file 7 — Mixing index shown in Fig. 5. [file 41565_2026_2164_MOESM7_ESM.zip › Source Data Fig. 5/Mixing index (Figure 5)/1to2to1_2arm/raw/20240411_Astem_2armlinker_121_40uMDFHBI_10nMHBC620_20-40-50_Sample3_4_cell1_cond2.tif]

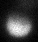

Supplement: Supplementary file 7 — Mixing index shown in Fig. 5. [file 41565_2026_2164_MOESM7_ESM.zip › Source Data Fig. 5/Mixing index (Figure 5)/2to1to2_2arm/raw/20240411_Astem_2armlinker_212_40uMDFHBI_10nMHBC620_20-40-50_Sample3_1_cell1_cond4.tif]

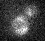

Supplement: Supplementary file 7 — Mixing index shown in Fig. 5. [file 41565_2026_2164_MOESM7_ESM.zip › Source Data Fig. 5/Mixing index (Figure 5)/2to1to2_4arm/raw/20240411_Astem_4armlinker_212_40uMDFHBI_10nMHBC620_20-40-50_Sample2_2_cell2_cond4.tif]

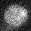

Supplement: Supplementary file 7 — Mixing index shown in Fig. 5. [file 41565_2026_2164_MOESM7_ESM.zip › Source Data Fig. 5/Mixing index (Figure 5)/2to1to2_4arm/raw/20240411_Astem_4armlinker_212_40uMDFHBI_10nMHBC620_20-40-50_Sample2_2_cell2_cond3.tif]

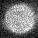

Supplement: Supplementary file 7 — Mixing index shown in Fig. 5. [file 41565_2026_2164_MOESM7_ESM.zip › Source Data Fig. 5/Mixing index (Figure 5)/1to3to1_2arm/raw/20240425_1to3to1_linker_2arm_sample2_40uMDFHBI_10nMHBC620_20_40_50_2_cell2_cond1.tif]

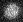

Supplement: Supplementary file 7 — Mixing index shown in Fig. 5. [file 41565_2026_2164_MOESM7_ESM.zip › Source Data Fig. 5/Mixing index (Figure 5)/2to1to2_4arm/raw/20240411_Astem_4armlinker_212_40uMDFHBI_10nMHBC620_20-40-50_Sample2_2_cell1_cond4.tif]

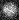

Supplement: Supplementary file 7 — Mixing index shown in Fig. 5. [file 41565_2026_2164_MOESM7_ESM.zip › Source Data Fig. 5/Mixing index (Figure 5)/2to1to2_4arm/raw/20240411_Astem_4armlinker_212_40uMDFHBI_10nMHBC620_20-40-50_Sample2_2_cell1_cond6.tif]

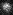

Supplement: Supplementary file 7 — Mixing index shown in Fig. 5. [file 41565_2026_2164_MOESM7_ESM.zip › Source Data Fig. 5/Mixing index (Figure 5)/1to3to1_2arm/raw/20240425_1to3to1_linker_2arm_sample3_40uMDFHBI_10nMHBC620_20_40_50_1_cell2_cond1.tif]

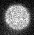

Supplement: Supplementary file 7 — Mixing index shown in Fig. 5. [file 41565_2026_2164_MOESM7_ESM.zip › Source Data Fig. 5/Mixing index (Figure 5)/1to3to1_2arm/raw/20240425_1to3to1_linker_2arm_sample2_40uMDFHBI_10nMHBC620_20_40_50_2_cell1_cond1.tif]

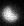

Supplement: Supplementary file 7 — Mixing index shown in Fig. 5. [file 41565_2026_2164_MOESM7_ESM.zip › Source Data Fig. 5/Mixing index (Figure 5)/1to2to1_2arm/raw/20240411_Astem_2armlinker_121_40uMDFHBI_10nMHBC620_20-40-50_Sample2_4_cell1_cond1.tif]

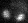

Supplement: Supplementary file 7 — Mixing index shown in Fig. 5. [file 41565_2026_2164_MOESM7_ESM.zip › Source Data Fig. 5/Mixing index (Figure 5)/2to1to2_2arm/raw/20240411_Astem_2armlinker_212_40uMDFHBI_10nMHBC620_20-40-50_Sample2_3_cell2_cond3.tif]

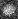

Supplement: Supplementary file 7 — Mixing index shown in Fig. 5. [file 41565_2026_2164_MOESM7_ESM.zip › Source Data Fig. 5/Mixing index (Figure 5)/2to1to2_4arm/raw/20240411_Astem_4armlinker_212_40uMDFHBI_10nMHBC620_20-40-50_Sample2_2_cell1_cond5.tif]

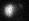

Supplement: Supplementary file 7 — Mixing index shown in Fig. 5. [file 41565_2026_2164_MOESM7_ESM.zip › Source Data Fig. 5/Mixing index (Figure 5)/2to1to2_2arm/raw/20240411_Astem_2armlinker_212_40uMDFHBI_10nMHBC620_20-40-50_Sample2_3_cell2_cond2.tif]

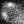

Supplement: Supplementary file 7 — Mixing index shown in Fig. 5. [file 41565_2026_2164_MOESM7_ESM.zip › Source Data Fig. 5/Mixing index (Figure 5)/1to2to1_2arm/raw/20240411_Astem_2armlinker_121_40uMDFHBI_10nMHBC620_20-40-50_Sample2_3_cell1_cond5.tif]

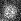

Supplement: Supplementary file 7 — Mixing index shown in Fig. 5. [file 41565_2026_2164_MOESM7_ESM.zip › Source Data Fig. 5/Mixing index (Figure 5)/2to1to2_4arm/raw/20240411_Astem_4armlinker_212_40uMDFHBI_10nMHBC620_20-40-50_Sample2_2_cell1_cond7.tif]

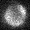

Supplement: Supplementary file 7 — Mixing index shown in Fig. 5. [file 41565_2026_2164_MOESM7_ESM.zip › Source Data Fig. 5/Mixing index (Figure 5)/1to3to1_2arm/raw/20240425_1to3to1_linker_2arm_sample3_40uMDFHBI_10nMHBC620_20_40_50_2_cell1_cond2.tif]

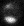

Supplement: Supplementary file 7 — Mixing index shown in Fig. 5. [file 41565_2026_2164_MOESM7_ESM.zip › Source Data Fig. 5/Mixing index (Figure 5)/1to2to1_2arm/raw/20240411_Astem_2armlinker_121_40uMDFHBI_10nMHBC620_20-40-50_Sample2_3_cell1_cond3.tif]

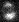

Supplement: Supplementary file 7 — Mixing index shown in Fig. 5. [file 41565_2026_2164_MOESM7_ESM.zip › Source Data Fig. 5/Mixing index (Figure 5)/1to3to1_2arm/raw/20240425_1to3to1_linker_2arm_sample3_40uMDFHBI_10nMHBC620_20_40_50_1_cell1_cond1.tif]

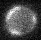

Supplement: Supplementary file 7 — Mixing index shown in Fig. 5. [file 41565_2026_2164_MOESM7_ESM.zip › Source Data Fig. 5/Mixing index (Figure 5)/1to3to1_2arm/raw/20240425_1to3to1_linker_2arm_sample3_40uMDFHBI_10nMHBC620_20_40_50_2_cell1_cond1.tif]

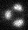

Supplement: Supplementary file 7 — Mixing index shown in Fig. 5. [file 41565_2026_2164_MOESM7_ESM.zip › Source Data Fig. 5/Mixing index (Figure 5)/2to1to2_2arm/raw/20240411_Astem_2armlinker_212_40uMDFHBI_10nMHBC620_20-40-50_Sample2_3_cell2_cond1.tif]

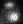

Supplement: Supplementary file 7 — Mixing index shown in Fig. 5. [file 41565_2026_2164_MOESM7_ESM.zip › Source Data Fig. 5/Mixing index (Figure 5)/1to2to1_2arm/raw/20240411_Astem_2armlinker_121_40uMDFHBI_10nMHBC620_20-40-50_Sample2_3_cell1_cond4.tif]

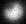

Supplement: Supplementary file 7 — Mixing index shown in Fig. 5. [file 41565_2026_2164_MOESM7_ESM.zip › Source Data Fig. 5/Mixing index (Figure 5)/1to3to1_2arm/raw/20240425_1to3to1_linker_2arm_sample3_40uMDFHBI_10nMHBC620_20_40_50_1_cell1_cond2.tif]

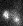

Supplement: Supplementary file 7 — Mixing index shown in Fig. 5. [file 41565_2026_2164_MOESM7_ESM.zip › Source Data Fig. 5/Mixing index (Figure 5)/2to1to2_2arm/raw/20240411_Astem_2armlinker_212_40uMDFHBI_10nMHBC620_20-40-50_Sample3_3_cell2_cond1.tif]

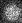

Supplement: Supplementary file 7 — Mixing index shown in Fig. 5. [file 41565_2026_2164_MOESM7_ESM.zip › Source Data Fig. 5/Mixing index (Figure 5)/2to1to2_4arm/raw/20240411_Astem_4armlinker_212_40uMDFHBI_10nMHBC620_20-40-50_Sample2_2_cell1_cond2.tif]

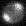

Supplement: Supplementary file 7 — Mixing index shown in Fig. 5. [file 41565_2026_2164_MOESM7_ESM.zip › Source Data Fig. 5/Mixing index (Figure 5)/1to3to1_2arm/raw/20240425_1to3to1_linker_2arm_sample1_40uMDFHBI_10nMHBC620_20_40_50_3_cell1_cond5.tif]

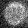

Supplement: Supplementary file 7 — Mixing index shown in Fig. 5. [file 41565_2026_2164_MOESM7_ESM.zip › Source Data Fig. 5/Mixing index (Figure 5)/1to3to1_2arm/raw/20240425_1to3to1_linker_2arm_sample2_40uMDFHBI_10nMHBC620_20_40_50_3_cell2_cond3.tif]

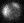

Supplement: Supplementary file 7 — Mixing index shown in Fig. 5. [file 41565_2026_2164_MOESM7_ESM.zip › Source Data Fig. 5/Mixing index (Figure 5)/1to3to1_2arm/raw/20240425_1to3to1_linker_2arm_sample1_40uMDFHBI_10nMHBC620_20_40_50_3_cell1_cond6.tif]

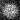

Supplement: Supplementary file 7 — Mixing index shown in Fig. 5. [file 41565_2026_2164_MOESM7_ESM.zip › Source Data Fig. 5/Mixing index (Figure 5)/2to1to2_4arm/raw/20240411_Astem_4armlinker_212_40uMDFHBI_10nMHBC620_20-40-50_Sample2_2_cell1_cond3.tif]

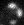

Supplement: Supplementary file 7 — Mixing index shown in Fig. 5. [file 41565_2026_2164_MOESM7_ESM.zip › Source Data Fig. 5/Mixing index (Figure 5)/2to1to2_2arm/raw/20240411_Astem_2armlinker_212_40uMDFHBI_10nMHBC620_20-40-50_Sample2_3_cell1_cond3.tif]

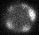

Supplement: Supplementary file 7 — Mixing index shown in Fig. 5. [file 41565_2026_2164_MOESM7_ESM.zip › Source Data Fig. 5/Mixing index (Figure 5)/1to3to1_2arm/raw/20240425_1to3to1_linker_2arm_sample1_40uMDFHBI_10nMHBC620_20_40_50_3_cell1_cond3.tif]

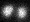

Supplement: Supplementary file 7 — Mixing index shown in Fig. 5. [file 41565_2026_2164_MOESM7_ESM.zip › Source Data Fig. 5/Mixing index (Figure 5)/2to1to2_2arm/raw/20240411_Astem_2armlinker_212_40uMDFHBI_10nMHBC620_20-40-50_Sample3_3_cell1_cond2.tif]

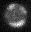

Supplement: Supplementary file 7 — Mixing index shown in Fig. 5. [file 41565_2026_2164_MOESM7_ESM.zip › Source Data Fig. 5/Mixing index (Figure 5)/1to3to1_2arm/raw/20240425_1to3to1_linker_2arm_sample1_40uMDFHBI_10nMHBC620_20_40_50_3_cell1_cond4.tif]

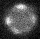

Supplement: Supplementary file 7 — Mixing index shown in Fig. 5. [file 41565_2026_2164_MOESM7_ESM.zip › Source Data Fig. 5/Mixing index (Figure 5)/1to3to1_2arm/raw/20240425_1to3to1_linker_2arm_sample1_40uMDFHBI_10nMHBC620_20_40_50_3_cell1_cond1.tif]

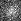

Supplement: Supplementary file 7 — Mixing index shown in Fig. 5. [file 41565_2026_2164_MOESM7_ESM.zip › Source Data Fig. 5/Mixing index (Figure 5)/2to1to2_4arm/raw/20240411_Astem_4armlinker_212_40uMDFHBI_10nMHBC620_20-40-50_Sample2_2_cell1_cond8.tif]

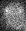

Supplement: Supplementary file 7 — Mixing index shown in Fig. 5. [file 41565_2026_2164_MOESM7_ESM.zip › Source Data Fig. 5/Mixing index (Figure 5)/2to1to2_4arm/raw/20240411_Astem_4armlinker_212_40uMDFHBI_10nMHBC620_20-40-50_Sample1_4_cell1_cond5.tif]

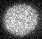

Supplement: Supplementary file 7 — Mixing index shown in Fig. 5. [file 41565_2026_2164_MOESM7_ESM.zip › Source Data Fig. 5/Mixing index (Figure 5)/2to1to2_4arm/raw/20240411_Astem_4armlinker_212_40uMDFHBI_10nMHBC620_20-40-50_Sample1_4_cell1_cond2.tif]

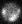

Supplement: Supplementary file 7 — Mixing index shown in Fig. 5. [file 41565_2026_2164_MOESM7_ESM.zip › Source Data Fig. 5/Mixing index (Figure 5)/1to2to1_2arm/raw/20240411_Astem_2armlinker_121_40uMDFHBI_10nMHBC620_20-40-50_Sample2_1_cell1_cond3.tif]

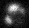

Supplement: Supplementary file 7 — Mixing index shown in Fig. 5. [file 41565_2026_2164_MOESM7_ESM.zip › Source Data Fig. 5/Mixing index (Figure 5)/2to1to2_2arm/raw/20240411_Astem_2armlinker_212_40uMDFHBI_10nMHBC620_20-40-50_Sample2_3_cell1_cond1.tif]

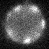

Supplement: Supplementary file 7 — Mixing index shown in Fig. 5. [file 41565_2026_2164_MOESM7_ESM.zip › Source Data Fig. 5/Mixing index (Figure 5)/1to3to1_2arm/raw/20240425_1to3to1_linker_2arm_sample1_40uMDFHBI_10nMHBC620_20_40_50_3_cell1_cond2.tif]

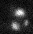

Supplement: Supplementary file 7 — Mixing index shown in Fig. 5. [file 41565_2026_2164_MOESM7_ESM.zip › Source Data Fig. 5/Mixing index (Figure 5)/2to1to2_2arm/raw/20240411_Astem_2armlinker_212_40uMDFHBI_10nMHBC620_20-40-50_Sample2_3_cell1_cond2.tif]

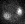

Supplement: Supplementary file 7 — Mixing index shown in Fig. 5. [file 41565_2026_2164_MOESM7_ESM.zip › Source Data Fig. 5/Mixing index (Figure 5)/1to2to1_2arm/raw/20240411_Astem_2armlinker_121_40uMDFHBI_10nMHBC620_20-40-50_Sample2_3_cell1_cond1.tif]

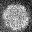

Supplement: Supplementary file 7 — Mixing index shown in Fig. 5. [file 41565_2026_2164_MOESM7_ESM.zip › Source Data Fig. 5/Mixing index (Figure 5)/2to1to2_4arm/raw/20240411_Astem_4armlinker_212_40uMDFHBI_10nMHBC620_20-40-50_Sample1_4_cell1_cond4.tif]

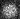

Supplement: Supplementary file 7 — Mixing index shown in Fig. 5. [file 41565_2026_2164_MOESM7_ESM.zip › Source Data Fig. 5/Mixing index (Figure 5)/2to1to2_4arm/raw/20240411_Astem_4armlinker_212_40uMDFHBI_10nMHBC620_20-40-50_Sample2_2_cell1_cond1.tif]

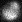

Supplement: Supplementary file 7 — Mixing index shown in Fig. 5. [file 41565_2026_2164_MOESM7_ESM.zip › Source Data Fig. 5/Mixing index (Figure 5)/1to2to1_2arm/raw/20240411_Astem_2armlinker_121_40uMDFHBI_10nMHBC620_20-40-50_Sample1_3_cell1_cond4.tif]

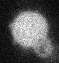

Supplement: Supplementary file 7 — Mixing index shown in Fig. 5. [file 41565_2026_2164_MOESM7_ESM.zip › Source Data Fig. 5/Mixing index (Figure 5)/1to2to1_4arm/raw/20240801_4arm_1to2to1_NucDFHBIHBC_20-40-50_Sample2_2_cell2_cond1.tif]

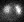

Supplement: Supplementary file 7 — Mixing index shown in Fig. 5. [file 41565_2026_2164_MOESM7_ESM.zip › Source Data Fig. 5/Mixing index (Figure 5)/1to2to1_2arm/raw/20240411_Astem_2armlinker_121_40uMDFHBI_10nMHBC620_20-40-50_Sample2_3_cell1_cond2.tif]

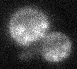

Supplement: Supplementary file 7 — Mixing index shown in Fig. 5. [file 41565_2026_2164_MOESM7_ESM.zip › Source Data Fig. 5/Mixing index (Figure 5)/2to1to2_4arm/raw/20240411_Astem_4armlinker_212_40uMDFHBI_10nMHBC620_20-40-50_Sample2_3_cell1_cond2.tif]

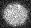

Supplement: Supplementary file 7 — Mixing index shown in Fig. 5. [file 41565_2026_2164_MOESM7_ESM.zip › Source Data Fig. 5/Mixing index (Figure 5)/2to1to2_4arm/raw/20240411_Astem_4armlinker_212_40uMDFHBI_10nMHBC620_20-40-50_Sample1_4_cell1_cond1.tif]

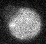

Supplement: Supplementary file 7 — Mixing index shown in Fig. 5. [file 41565_2026_2164_MOESM7_ESM.zip › Source Data Fig. 5/Mixing index (Figure 5)/2to1to2_4arm/raw/20240411_Astem_4armlinker_212_40uMDFHBI_10nMHBC620_20-40-50_Sample2_3_cell1_cond1.tif]

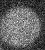

Supplement: Supplementary file 7 — Mixing index shown in Fig. 5. [file 41565_2026_2164_MOESM7_ESM.zip › Source Data Fig. 5/Mixing index (Figure 5)/2to1to2_4arm/raw/20240411_Astem_4armlinker_212_40uMDFHBI_10nMHBC620_20-40-50_Sample3_2_cell1_cond1.tif]

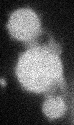

Supplement: Supplementary file 7 — Mixing index shown in Fig. 5. [file 41565_2026_2164_MOESM7_ESM.zip › Source Data Fig. 5/Mixing index (Figure 5)/1to2to1_4arm/raw/20240801_4arm_1to2to1_NucDFHBIHBC_20-40-50_Sample2_2_cell2_cond2.tif]

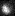

Supplement: Supplementary file 7 — Mixing index shown in Fig. 5. [file 41565_2026_2164_MOESM7_ESM.zip › Source Data Fig. 5/Mixing index (Figure 5)/1to2to1_2arm/raw/20240411_Astem_2armlinker_121_40uMDFHBI_10nMHBC620_20-40-50_Sample1_2_cell1_cond5.tif]

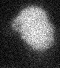

Supplement: Supplementary file 7 — Mixing index shown in Fig. 5. [file 41565_2026_2164_MOESM7_ESM.zip › Source Data Fig. 5/Mixing index (Figure 5)/1to2to1_4arm/raw/20240801_4arm_1to2to1_NucDFHBIHBC_20-40-50_Sample3_3_cell2_cond2.tif]

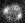

Supplement: Supplementary file 7 — Mixing index shown in Fig. 5. [file 41565_2026_2164_MOESM7_ESM.zip › Source Data Fig. 5/Mixing index (Figure 5)/1to2to1_2arm/raw/20240411_Astem_2armlinker_121_40uMDFHBI_10nMHBC620_20-40-50_Sample1_3_cell1_cond2.tif]

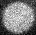

Supplement: Supplementary file 7 — Mixing index shown in Fig. 5. [file 41565_2026_2164_MOESM7_ESM.zip › Source Data Fig. 5/Mixing index (Figure 5)/2to1to2_4arm/raw/20240411_Astem_4armlinker_212_40uMDFHBI_10nMHBC620_20-40-50_Sample1_4_cell1_cond3.tif]

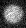

Supplement: Supplementary file 7 — Mixing index shown in Fig. 5. [file 41565_2026_2164_MOESM7_ESM.zip › Source Data Fig. 5/Mixing index (Figure 5)/1to2to1_4arm/raw/20240801_4arm_1to2to1_NucDFHBIHBC_20-40-50_Sample3_3_cell2_cond1.tif]

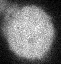

Supplement: Supplementary file 7 — Mixing index shown in Fig. 5. [file 41565_2026_2164_MOESM7_ESM.zip › Source Data Fig. 5/Mixing index (Figure 5)/1to2to1_4arm/raw/20240801_4arm_1to2to1_NucDFHBIHBC_20-40-50_Sample3_2_cell3_cond1.tif]

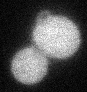

Supplement: Supplementary file 7 — Mixing index shown in Fig. 5. [file 41565_2026_2164_MOESM7_ESM.zip › Source Data Fig. 5/Mixing index (Figure 5)/1to2to1_4arm/raw/20240801_4arm_1to2to1_NucDFHBIHBC_20-40-50_Sample2_2_cell3_cond1.tif]

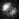

Supplement: Supplementary file 7 — Mixing index shown in Fig. 5. [file 41565_2026_2164_MOESM7_ESM.zip › Source Data Fig. 5/Mixing index (Figure 5)/1to2to1_2arm/raw/20240411_Astem_2armlinker_121_40uMDFHBI_10nMHBC620_20-40-50_Sample1_2_cell1_cond9.tif]

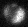

Supplement: Supplementary file 7 — Mixing index shown in Fig. 5. [file 41565_2026_2164_MOESM7_ESM.zip › Source Data Fig. 5/Mixing index (Figure 5)/1to2to1_2arm/raw/20240411_Astem_2armlinker_121_40uMDFHBI_10nMHBC620_20-40-50_Sample1_3_cell1_cond3.tif]

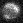

Supplement: Supplementary file 7 — Mixing index shown in Fig. 5. [file 41565_2026_2164_MOESM7_ESM.zip › Source Data Fig. 5/Mixing index (Figure 5)/1to2to1_2arm/raw/20240411_Astem_2armlinker_121_40uMDFHBI_10nMHBC620_20-40-50_Sample1_2_cell1_cond8.tif]

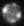

Supplement: Supplementary file 7 — Mixing index shown in Fig. 5. [file 41565_2026_2164_MOESM7_ESM.zip › Source Data Fig. 5/Mixing index (Figure 5)/1to2to1_2arm/raw/20240411_Astem_2armlinker_121_40uMDFHBI_10nMHBC620_20-40-50_Sample2_1_cell1_cond2.tif]

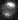

Supplement: Supplementary file 7 — Mixing index shown in Fig. 5. [file 41565_2026_2164_MOESM7_ESM.zip › Source Data Fig. 5/Mixing index (Figure 5)/1to2to1_2arm/raw/20240411_Astem_2armlinker_121_40uMDFHBI_10nMHBC620_20-40-50_Sample1_2_cell1_cond4.tif]

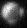

Supplement: Supplementary file 7 — Mixing index shown in Fig. 5. [file 41565_2026_2164_MOESM7_ESM.zip › Source Data Fig. 5/Mixing index (Figure 5)/1to2to1_2arm/raw/20240411_Astem_2armlinker_121_40uMDFHBI_10nMHBC620_20-40-50_Sample1_3_cell1_cond1.tif]

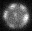

Supplement: Supplementary file 7 — Mixing index shown in Fig. 5. [file 41565_2026_2164_MOESM7_ESM.zip › Source Data Fig. 5/Mixing index (Figure 5)/1to2to1_2arm/raw/20240411_Astem_2armlinker_121_40uMDFHBI_10nMHBC620_20-40-50_Sample2_1_cell1_cond1.tif]

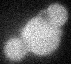

Supplement: Supplementary file 7 — Mixing index shown in Fig. 5. [file 41565_2026_2164_MOESM7_ESM.zip › Source Data Fig. 5/Mixing index (Figure 5)/1to2to1_4arm/raw/20240801_4arm_1to2to1_NucDFHBIHBC_20-40-50_Sample3_1_cell1_cond2.tif]

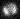

Supplement: Supplementary file 7 — Mixing index shown in Fig. 5. [file 41565_2026_2164_MOESM7_ESM.zip › Source Data Fig. 5/Mixing index (Figure 5)/1to2to1_2arm/raw/20240411_Astem_2armlinker_121_40uMDFHBI_10nMHBC620_20-40-50_Sample1_2_cell1_cond6.tif]

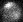

Supplement: Supplementary file 7 — Mixing index shown in Fig. 5. [file 41565_2026_2164_MOESM7_ESM.zip › Source Data Fig. 5/Mixing index (Figure 5)/1to2to1_2arm/raw/20240411_Astem_2armlinker_121_40uMDFHBI_10nMHBC620_20-40-50_Sample1_2_cell1_cond2.tif]

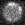

Supplement: Supplementary file 7 — Mixing index shown in Fig. 5. [file 41565_2026_2164_MOESM7_ESM.zip › Source Data Fig. 5/Mixing index (Figure 5)/1to2to1_2arm/raw/20240411_Astem_2armlinker_121_40uMDFHBI_10nMHBC620_20-40-50_Sample1_2_cell1_cond7.tif]

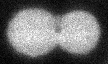

Supplement: Supplementary file 7 — Mixing index shown in Fig. 5. [file 41565_2026_2164_MOESM7_ESM.zip › Source Data Fig. 5/Mixing index (Figure 5)/1to2to1_4arm/raw/20240801_4arm_1to2to1_NucDFHBIHBC_20-40-50_Sample3_3_cell1_cond1.tif]

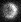

Supplement: Supplementary file 7 — Mixing index shown in Fig. 5. [file 41565_2026_2164_MOESM7_ESM.zip › Source Data Fig. 5/Mixing index (Figure 5)/1to2to1_2arm/raw/20240411_Astem_2armlinker_121_40uMDFHBI_10nMHBC620_20-40-50_Sample1_1_cell1_cond3.tif]

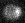

Supplement: Supplementary file 7 — Mixing index shown in Fig. 5. [file 41565_2026_2164_MOESM7_ESM.zip › Source Data Fig. 5/Mixing index (Figure 5)/1to2to1_2arm/raw/20240411_Astem_2armlinker_121_40uMDFHBI_10nMHBC620_20-40-50_Sample1_1_cell1_cond2.tif]

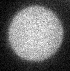

Supplement: Supplementary file 7 — Mixing index shown in Fig. 5. [file 41565_2026_2164_MOESM7_ESM.zip › Source Data Fig. 5/Mixing index (Figure 5)/1to2to1_4arm/raw/20240801_4arm_1to2to1_NucDFHBIHBC_20-40-50_Sample1_4_cell1_cond1.tif]

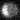

Supplement: Supplementary file 7 — Mixing index shown in Fig. 5. [file 41565_2026_2164_MOESM7_ESM.zip › Source Data Fig. 5/Mixing index (Figure 5)/1to2to1_2arm/raw/20240411_Astem_2armlinker_121_40uMDFHBI_10nMHBC620_20-40-50_Sample1_1_cell1_cond4.tif]

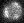

Supplement: Supplementary file 7 — Mixing index shown in Fig. 5. [file 41565_2026_2164_MOESM7_ESM.zip › Source Data Fig. 5/Mixing index (Figure 5)/1to2to1_2arm/raw/20240411_Astem_2armlinker_121_40uMDFHBI_10nMHBC620_20-40-50_Sample1_1_cell1_cond7.tif]

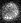

Supplement: Supplementary file 7 — Mixing index shown in Fig. 5. [file 41565_2026_2164_MOESM7_ESM.zip › Source Data Fig. 5/Mixing index (Figure 5)/1to2to1_2arm/raw/20240411_Astem_2armlinker_121_40uMDFHBI_10nMHBC620_20-40-50_Sample1_1_cell1_cond6.tif]

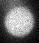

Supplement: Supplementary file 7 — Mixing index shown in Fig. 5. [file 41565_2026_2164_MOESM7_ESM.zip › Source Data Fig. 5/Mixing index (Figure 5)/1to2to1_4arm/raw/20240801_4arm_1to2to1_NucDFHBIHBC_20-40-50_Sample3_2_cell1_cond2.tif]

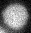

Supplement: Supplementary file 7 — Mixing index shown in Fig. 5. [file 41565_2026_2164_MOESM7_ESM.zip › Source Data Fig. 5/Mixing index (Figure 5)/1to2to1_4arm/raw/20240801_4arm_1to2to1_NucDFHBIHBC_20-40-50_Sample1_4_cell2_cond2.tif]

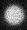

Supplement: Supplementary file 7 — Mixing index shown in Fig. 5. [file 41565_2026_2164_MOESM7_ESM.zip › Source Data Fig. 5/Mixing index (Figure 5)/1to2to1_4arm/raw/20240801_4arm_1to2to1_NucDFHBIHBC_20-40-50_Sample3_2_cell2_cond3.tif]

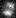

Supplement: Supplementary file 7 — Mixing index shown in Fig. 5. [file 41565_2026_2164_MOESM7_ESM.zip › Source Data Fig. 5/Mixing index (Figure 5)/1to2to1_2arm/raw/20240411_Astem_2armlinker_121_40uMDFHBI_10nMHBC620_20-40-50_Sample1_2_cell1_cond1.tif]

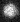

Supplement: Supplementary file 7 — Mixing index shown in Fig. 5. [file 41565_2026_2164_MOESM7_ESM.zip › Source Data Fig. 5/Mixing index (Figure 5)/1to2to1_4arm/raw/20240801_4arm_1to2to1_NucDFHBIHBC_20-40-50_Sample3_2_cell2_cond1.tif]

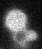

Supplement: Supplementary file 7 — Mixing index shown in Fig. 5. [file 41565_2026_2164_MOESM7_ESM.zip › Source Data Fig. 5/Mixing index (Figure 5)/1to2to1_4arm/raw/20240801_4arm_1to2to1_NucDFHBIHBC_20-40-50_Sample1_3_cell2_cond3.tif]

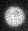

Supplement: Supplementary file 7 — Mixing index shown in Fig. 5. [file 41565_2026_2164_MOESM7_ESM.zip › Source Data Fig. 5/Mixing index (Figure 5)/1to2to1_4arm/raw/20240801_4arm_1to2to1_NucDFHBIHBC_20-40-50_Sample1_3_cell2_cond2.tif]

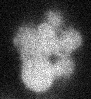

Supplement: Supplementary file 7 — Mixing index shown in Fig. 5. [file 41565_2026_2164_MOESM7_ESM.zip › Source Data Fig. 5/Mixing index (Figure 5)/1to2to1_4arm/raw/20240801_4arm_1to2to1_NucDFHBIHBC_20-40-50_Sample2_2_cell1_cond1.tif]

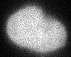

Supplement: Supplementary file 7 — Mixing index shown in Fig. 5. [file 41565_2026_2164_MOESM7_ESM.zip › Source Data Fig. 5/Mixing index (Figure 5)/1to2to1_4arm/raw/20240801_4arm_1to2to1_NucDFHBIHBC_20-40-50_Sample3_2_cell1_cond1.tif]

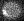

Supplement: Supplementary file 7 — Mixing index shown in Fig. 5. [file 41565_2026_2164_MOESM7_ESM.zip › Source Data Fig. 5/Mixing index (Figure 5)/1to2to1_2arm/raw/20240411_Astem_2armlinker_121_40uMDFHBI_10nMHBC620_20-40-50_Sample1_1_cell1_cond8.tif]

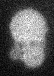

Supplement: Supplementary file 7 — Mixing index shown in Fig. 5. [file 41565_2026_2164_MOESM7_ESM.zip › Source Data Fig. 5/Mixing index (Figure 5)/1to2to1_4arm/raw/20240801_4arm_1to2to1_NucDFHBIHBC_20-40-50_Sample2_1_cell2_cond1.tif]

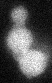

Supplement: Supplementary file 7 — Mixing index shown in Fig. 5. [file 41565_2026_2164_MOESM7_ESM.zip › Source Data Fig. 5/Mixing index (Figure 5)/1to2to1_4arm/raw/20240801_4arm_1to2to1_NucDFHBIHBC_20-40-50_Sample1_1_cell1_cond2.tif]

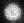

Supplement: Supplementary file 7 — Mixing index shown in Fig. 5. [file 41565_2026_2164_MOESM7_ESM.zip › Source Data Fig. 5/Mixing index (Figure 5)/1to2to1_4arm/raw/20240801_4arm_1to2to1_NucDFHBIHBC_20-40-50_Sample1_3_cell2_cond1.tif]

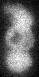

Supplement: Supplementary file 7 — Mixing index shown in Fig. 5. [file 41565_2026_2164_MOESM7_ESM.zip › Source Data Fig. 5/Mixing index (Figure 5)/1to2to1_4arm/raw/20240801_4arm_1to2to1_NucDFHBIHBC_20-40-50_Sample3_2_cell2_cond2.tif]
